# Frequency of hybridization between *Ostrinia nubilalis E*-and *Z*-pheromone races in regions of sympatry within the United States

**DOI:** 10.1002/ece3.639

**Published:** 2013-06-24

**Authors:** Brad S Coates, Holly Johnson, Kyung-Seok Kim, Richard L Hellmich, Craig A Abel, Charles Mason, Thomas W Sappington

**Affiliations:** 1 USDA-ARS, Corn Insects and Crop Genetics Research Unit, Genetics Laboratory, Iowa State UniversityAmes, Iowa, 50011; 2 Department of Entomology, Iowa State UniversityAmes, Iowa, 50011; 3 Entomology and Wildlife Ecology, University of Delaware531 S College Ave RM 250, Newark, Delaware, 19716-2160

**Keywords:** Gene flow, hybridization, pheromone variation, reproductive isolation

## Abstract

Female European corn borer, *Ostrinia nubilalis*, produce and males respond to sex pheromone blends with either *E*- or *Z*-Δ11-tetradecenyl acetate as the major component. *E*- and *Z*-race populations are sympatric in the Eastern United States, Southeastern Canada, and the Mediterranean region of Europe. The *E*- and *Z*-pheromone races of *O. nubilalis* are models for incipient species formation, but hybridization frequencies within natural populations remain obscure due to lack of a high-throughput phenotyping method. Lassance et al. previously identified a pheromone gland-expressed fatty-acyl reductase gene (*pgfar*) that controls the ratio of Δ11-tetradecenyl acetate stereoisomers. We identified three single nucleotide polymorphism (SNP) markers within *pgfar* that are differentially fixed between *E*- and *Z*-race females, and that are ≥98.2% correlated with female pheromone ratios measured by gas chromatography. Genotypic data from locations in the United States demonstrated that *pgfar*-z alleles were fixed within historically allopatric *Z*-pheromone race populations in the Midwest, and that hybrid frequency ranged from 0.00 to 0.42 within 11 sympatric sites where the two races co-occur in the Eastern United States (mean hybridization frequency or heterozygosity (*H*_O_) = 0.226 ± 0.279). Estimates of hybridization between the *E*- and *Z*-races are important for understanding the dynamics involved in maintaining race integrity, and are consistent with previous estimates of low levels of genetic divergence between *E*- and *Z*-races and the presence of weak prezygotic mating barriers.

This work describes the development of new single nucleotide polymorphism (SNP) markers within the pheromone gland expressed fatty acyl reductase (*pgfar*) gene of *Ostrinia nubilalis*. These SNPs were shown to segregate based upon female pheromone production, and thus provide the first description of an assay for genetic determination of *O. nubilalis* pheromone strain from field-collected samples. These assays were applied to estimate hybridization within field populations, and represent valuable tools for future population genetic studies of this species.

## Introduction

Biological traits that influence reproductive biology, chemical communication, and mate attraction can influence gene flow within a species, maintain phenotypic diversity among ecotypes, and may ultimately contribute to sympatric speciation. Key mutations that establish prezygotic mating barriers effectively decrease gene flow within previously panmictic populations and allow the subsequent accumulation of divergent life history traits (Roelofs and Rooney 51). During the early stages of diversification, incipient species often maintain high levels of gene flow, such that introgression occurs in regions of the genome not linked to genes directly involved in speciation (Wu 66; Lassance et al. 35). Incipient species can retain genomic regions with shared ancestral polymorphisms, as well as regions with derived mutations at a few key loci that are fixed differentially among divergent lineages (Gentile et al. 20; Machado et al. 41; Roelofs and Rooney 51). Incomplete barriers to gene flow and consequent introgression can lead to homogenization of genomic regions not linked to genes involved in speciation. Because genetic drift and selection can lead to divergent molecular signals and phenotypic traits unrelated to the mechanisms involved in speciation, analyses of incipient species provide not only opportunities, but unique challenges for the study of contemporary speciation.

The European corn borer, *Ostrinia nubilalis*, is a polyphagous lepidopteran insect native to Eastern Europe and Western Asia, but was inadvertently introduced to North America in the early 1900s (Vinal 64). Phenotypic variation includes females that produce, and males that respond to, pheromone blends of 99:1 or 3:97 *E*-: *Z*-Δ11-tetradecenyl acetate (*E*11- and *Z*11-14:OAc) (Klun and Brindley 30; Kochansky et al. 32; Roelofs et al. 77). The geographic distribution of *O. nubilalis* that dominantly use the *Z*11-14:OAc isomer for sexual communication (*Z*-race) extend across the eastern two thirds of the United States, southeastern Canada, and Europe (Klun and Cooperators 29). In contrast, populations of the *E*-race are restricted to the Eastern United States (O'Rourke et al. 49), southeastern Canada (J. Smith, unpubl. data), and the Mediterranean region of Europe (Anglade and Stockel 1). Limited gene flow between *O. nubilalis* pheromone races has been suggested from molecular analyses (Harrison and Vawter 75; Cardé et al. 69; Cianchi et al. 70; Glover et al. 22; Dopman et al. 74), laboratory choice tests (Liebherr and Roelofs 36), and field collections of F_1_ hybrid females that produce a 65:35 *E*11- to *Z*11-14:OAc ratio (Klun and Maini 31; Roelofs et al. 52; Durant et al. 15).

The female *O. nubilalis* pheromone gland is comprised of a single layer of epidermal cells located in the 8th and fused 9th/10th abdominal segments (Ma and Roelofs 40). Both races produce an approximately equal proportion of *E*- and *Z*-11-tetradecenyl, the precursors of *E*11- and *Z*11-14:OAc (Roelofs et al. 53, 54). The specific *E*11 to *Z*11-14:OAc isomer ratios produced by female *E*- and *Z*-race *O. nubilalis* is controlled by a single codominant genetic locus (Klun and Maini 31; Roelofs et al. 53; Dopman et al. 12), but are modified to a lesser degree by unlinked genetic loci that were revealed by analyses of backcross progeny (Löfstedt et al. 39; Zhu et al. 67). Subsequent genetic linkage analysis indicated that the major locus controlling production of the *E*- or *Z*-isomer mapped to the pheromone gland fatty-acyl reductase gene, *pgfar* (Lassance et al. 35), and that two alleles, *pgfar-e* and *pgfar-z*, are differentially expressed in the pheromone gland of *E*- and *Z*-race females, respectively. Any barrier to gene flow between *O. nubilalis E*- and *Z*-race field populations may be reinforced by a reduced ability of F_1_ males to locate females of either race (Glover et al. 22), which suggests a coevolution between male and female reproductive traits (Lassance 33). Despite the known association between *pgfar* and pheromone isomer production, questions remain regarding this relationship in natural populations and the level of intrarace variation at the *pgfar* locus (Lassance 33).

The degree of gene flow in a hybrid zone can be interpreted as a measure of species divergence, and can be important for understanding speciation mechanisms. Female *O. nubilalis* pheromones (phenotypes) can be determined via separation of pheromone gland-produced hydrocarbons by gas chromatography (GC) analysis and comparison to synthetic standards. GC analyses are often difficult to apply within large population studies because the pheromone gland must be dissected from virgin females during scotophase when the pheromone titer is highest (Smith et al. 58). Male *O. nubilalis* can be collected in traps baited with either the synthetic *E*- or *Z*-pheromone blend, but the lures show reduced fidelity to race when imprecise formulations are used (Bartels et al. 2; Mason et al. 44; Pelozuelo and Frerot 76). Furthermore, identifying hybrid males captured in traps baited with either kind of lure requires GC analysis of the pheromone produced by female offspring from controlled backcrosses, a slow and logistically challenging undertaking. A fast and accurate molecular diagnostic assay would be of great value to those seeking to phenotype an individual's race, not only in studies of race interactions and genetic isolation, but in any ecological or behavioral study of this species conducted in areas of sympatry.

A single nucleotide polymorphism (SNP) is a single base substitution at a genomic locus, and SNPs are useful tools for population and genetic mapping studies (Glaubitz et al. 21). SNPs can be detected by automated genotyping assays (Tsuchihashi and Dracopoli 62) or low-throughput methods (Vignal et al. 78). Molecular genetic markers within or linked to genes affected by a recent selective sweep can be associated with divergent traits, and thus used to predict individual phenotypes in natural environments (Schulze and McMahon 55). In this study, we identified SNP markers in *O. nubilalis* associated with *pgfar-*e and *pgfar-z* alleles previously provided by work by Lassance et al. (35), and verified their pheromone race specificity by direct comparison to pheromone phenotypes determined by GC analyses. We then developed and applied a SNP assay to estimate rates of hybridization between *E*- and *Z*-pheromone races at several locations in the Eastern United States.

## Material and Methods

### Development of pheromone race-specific molecular genetic markers

GenBank nr database accessions GU808256 to GU808276 originally submitted by Lassance et al. (35) which contain cDNA sequences derived from the *O. nubilalis pgfar* gene were downloaded in FASTA format, and aligned using the MEGA 5.0 DNA sequence alignment utility (Tamura et al. 61) using the default parameters of the ClustalW algorithm (gap opening penalty 15, gap extension penalty 6.66, weight matrix IUB, and transition weight of 0.5). Nucleotide diversity (*d*) was estimated using MEGA 5.0 (Tamura et al. 61), and 100-bp sliding windows were iterated across the aligned cDNA sequences in 25-bp increments using Python scripts of DNAux 3.0 (http://www.portugene.com/software.html), and *Tajima's D* was estimated from each window using MEGA 5.0 (Tamura et al. 61). The sliding window analysis closely approximated that previously provided by Lassance et al. (35), but was replicated within this study to accurately determine the position of SNPs.

**Figure 1:**
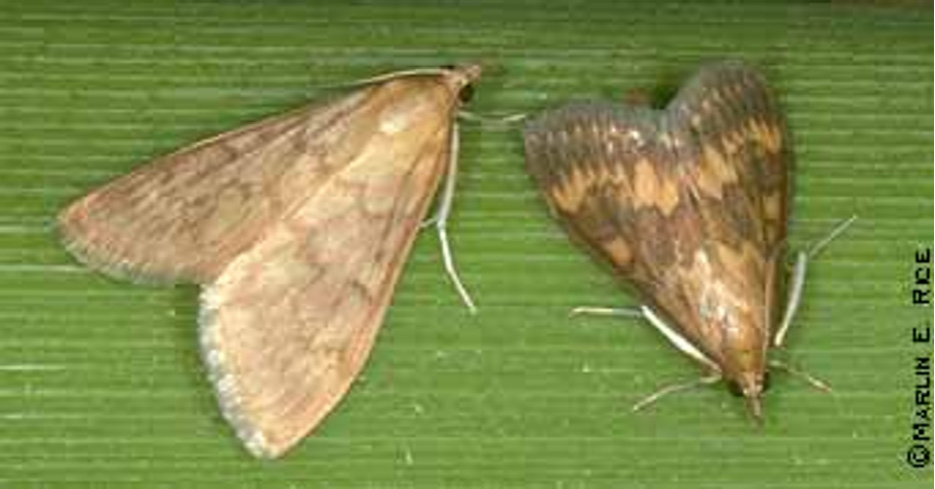
Adult *Ostrinia nubilalis* moths (female [left] and male [right]) from the United States population. Photograph by Marlin E. Rice.

**Figure 2:**
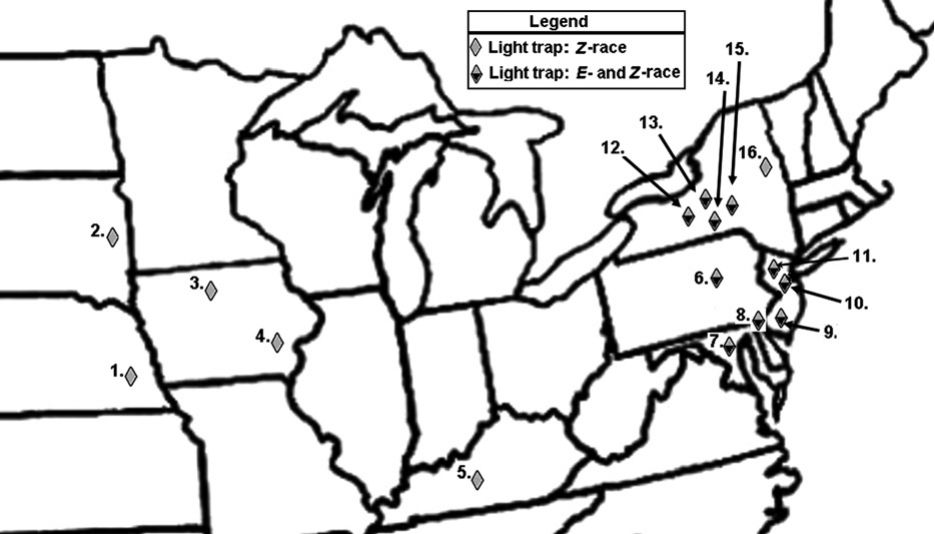
Map of *Ostrinia nubilalis* sample collection sites from infested regions of North America. Approximate geographic region of sympatric *E*- and *Z*-race populations that use a pheromone blend of predominantly *E*- or *Z*-Δ11-tetradecenyl acetate, respectively, are indicated by the shaded area along the East Coast of the United States. Locations 1 to 5 are in geographic regions where historically only the *Z*-race has been found, whereas locations 6 to 19 are in regions of sympatry.

Fixed sequence variation was identified between aligned *pgfar* cDNA sequences derived from *E*- and *Z*-pheromone races, and oligonucleotide primer pairs were designed from conserved flanking regions using Primer3Plus (Untergasser et al. 63). Specifically, oligonucleotide primer pairs were designed to PCR-amplify products containing *pgfar-e* and *pgfar-z* specific SNPs. Adult *O. nubilalis* (Fig. 1.) were collected by light trap at Crawfordsville, IA (*n* = 24), located in the Midwest region of the United States, where only *Z*-race populations are present (Table 1; Fig. 2). Samples from a laboratory colony of bivoltine *E*-race *O. nubilalis*, BENY, were obtained from C. Linn (Cornell University, Ithaca, NY). All DNA was extracted from the thorax of individual *O. nubilalis* adults as described by Coates and Hellmich (71), quantified on a NanoDrop 2000 (Thermo Scientific, Wilmington, DE), and diluted to 10 ng/μL with nuclease free water.

**Table 1:** Estimates of hybridization of *Ostrinia nubilalis E*- and *Z*-race within field populations in the United States

Site ID	Collection site	State	*n*	*H*_O_ among SNP markers							*H*_E_ (all SNPs)	*P*-value (all SNPs)
			M/F	*Taq*I	*Nde*II	*Mse*I						
1	Mead	NE	24/24	0.000	0.000	0.000	Monomorphic								
2	Brookings	SD	12/12	0.000	0.000	0.000	Monomorphic								
3	Kanawha	IA	24/24	0.000	0.000	0.000	Monomorphic								
4	Crawfordsville	IA	24/24	0.000	0.000	0.000	Monomorphic								
5	Lexington	KY	10/14	0.000	0.000	0.000	Monomorphic								
6	Snyder Co.	PA	13/11	0.083	0.083	0.083	0.156	0.126
7	Beltsville	MD	17/14	0.419	0.419	0.419	0.337	0.295
8	Newark	DE	44/44	0.193	0.193	0.193	0.347	<0.001^1^
9	Cohansey	NJ	24/24	0.125	0.125	0.125	0.281	<0.001^1^
10	Sergentsville	NJ	11/13	0.208	0.208	0.208	0.311	0.152
11	Little York	NJ	24/24	0.125	0.125	0.125	0.118	1.000
12	Yates	NY	3/7	0.200	0.200	0.200	0.506	0.082
13	Aurora	NY	10/7	0.059	0.059	0.059	0.059	1.000
14	Spencer	NY	9/11	0.100	0.100	0.100	0.097	1.000
15	Freeville	NY	5/6	0.182	0.182	0.182	0.173	1.000
16	Hartford	NY	6/4	0.000	0.000	0.000	Monomorphic								
C	BENY colony^2^	NY	44	0.000	0.000	0.000	Monomorphic								

Observed and expected heterozygosity at diagnostic *pgfar* SNP loci assayed by PCR-RFLP from *Ostrinia nubilalis* adults collected by light traps at indicated locations. Site IDs correspond to those on the map in Figure 1. BENY colony adults are included as a control for monomorphic *E*-race. NA = not applicable.

1 Significant at 0.05 level.

2 Not included in pairwise *F*_ST_ analyses.

SNPs within the *pgfar* gene were detected by three independent polymerase chain reaction-restriction fragment length polymorphism (PCR-RFLP) assays. DNA samples were amplified in 10-μL PCR solutions that included 1× Thermal polymerase buffer (Promega, Madison, WI), 2.5 mmol/L MgCl_2_, 50 μmol/L dNTPs, 20 ng DNA, 1.5 pmol of each primer (Table 2), and 0.3125 U Go*Taq* DNA polymerase (Promega). PCR was conducted in a Tetrad2 thermocycler (BioRad, Hercules, CA) programmed for 96°C for 3 min, followed by seven initial touchdown (TD) cycles consisting of 96°C for 20 sec, 65°C for 30 sec (–2°C/cycle each subsequent cycle), and 72°C for 30 sec. Final PCR amplification took place with 32 cycles of 96°C for 20 sec, 52°C for 30 sec, and 72°C for 30 sec. PCR product in the entire reaction volume was digested with a restriction endonuclease (RE) by adding 2.0 μL 10× buffer and 0.5 U of *Mse*I (New England BioLabs, Ipswich, MA), or *Nde*II or *Taq*I (Promega) in a 20-μL total reaction volume (Table 2), then incubated at 37°C (or 65°C for *Taq*I) for 14 h. Entire RE digest reaction volumes were loaded into 10-cm 3% agarose gels and separated at 80 V for 2 h. Resulting fragment sizes were estimated by comparison to a 50-bp ladder (Promega), and compared to those predicted for *pgfar-e* and *pgfar-z* alleles (Table 2). Differences between the number of observed and expected genotypes at Crawfordsville, IA (historically known to be *Z*-race) and BENY colony individuals (pure *E*-race colony provided by Dr. C. Linn) were tested for significance with Chi-square (χ^2^) tests. Expected heterozygote frequencies for the Crawfordsville, IA and BENY samples were calculated using Arlequin 3.5.1.2 (Excoffier and Lischer 16), and were performed as an initial test of allelic variation among groups of individuals with an a priori known phenotype (*E*- or *Z*-race).

**Table 2:** Oligonucleotide primer pairs that anneal within the pheromone gland fatty-acyl reductase, *pgfar*, gene of *Ostrinia nubilalis* to prime the PCR amplification of regions that contain pheromone race-specific single nucleotide polymorphisms (SNPs)

Name	Oligonucleotide primer	SNP	RE	Allele specific
pgFAR-tf	5′-TTC GAT TCG GGA ACC CAT A-3′	T^857^	*Taq*I(+)	*pgfar-z*
pgFAR-tr	5′-AGG TTC GCA ACG TGG TCT AC-3′	G^857^	*Taq*I(–)	*pgfar-e*
pgFAR-mnf	5′-G GGC AAC AAA GGA GTC AAG GT-3′	T^995^	*Nde*II(+)	*pgfar-e*
pgFAR-mnr	5′-CC AAA ATA TTT CCT GTA TTT TAW GCA-3′	G^995^	*Nde*II(–)	*pgfar-z*
pgFAR-mnf	5′-G GGC AAC AAA GGA GTC AAG GT-3′	T^1005^	*Mse*I(+)	*pgfar-e*
pgFAR-mnr	5′-CC AAA ATA TTT CCT GTA TTT TAW GCA-3′	G^1005^	*Mse*I(–)	*pgfar-z*

The SNPs are indicated as the nucleotide position within the *pgfar* cDNA and alternate nucleotides present at the locus (nucleotide ^position^), and the restriction enzyme (RE) used to detect each allele are indicated. G/T^995^ and G/T^1005^ are detected by PCR-RFLP of the same PCR-amplified fragment.

*Taq*I(–) 150 bp, *Taq*I(+) 118 + 32 bp; *Nde*II(–) 145 bp, *Nde*II(+) 83 + 62 bp; *Mse*I(–) 145 bp, *Mse*I(+) 93 + 52 bp.

### Correlation between genotype and pheromone production

To test the fidelity of the *pgfar* SNP markers for discriminating *O. nubilalis* females producing *E*-, *Z*-, and hybrid pheromone blends, both SNP genotype and GC-analyzed pheromone component data were collected from individual F_4_ females derived from six independent intercrossed families. Specifically, Families 1 to 6 were each initiated by pooling ~200 adults from a laboratory colony of pure bivoltine *Z*-race *O. nubilalis* maintained at the USDA-ARS, Corn Insects and Crop Genetics Research Unit, Ames, IA, and ~200 adults from the BENY colony (pure bivoltine *E*-race). The BENY and USDA colonies served as controls. All families were maintained as random mating populations of ≥1000 individuals until the F_4_ generation. Individual female F_4_ pupae were placed in 28-mL plastic cups along with a piece of moistened cotton dental wick and allowed to emerge as adults in growth chambers at 25°C and 16:8 (L:D). Pheromone glands were removed and volatile compounds extracted using methods similar to Durant et al. (15). In brief, pheromone ring glands were excised with micro-scissors at the terminal segment just anterior of the ring gland, during the 6th hour of scotophase (time of peak pheromone recovery, C. Mason, pers. comm.) from adult females the second day after eclosion (24–48 h old). Each gland was placed into a 50-μL point-tipped auto-sampler vial containing 5 μL of heptane and an internal standard of 4.5 ng cis-7-tetradecenyl acetate (7-TDA). 0053amples were held for ≥30 min at room temperature or stored in a −20°C freezer before analysis. DNA was extracted from the thorax of the same females, quantified, and diluted as described above. DNA samples were processed for genotyping at the *pgfar* PCR-RFLP marker loci using the restriction endonucleases *Taq*I, *Nde*II, and *Mse*I as described above.

A 10-μL Varian 8200 auto-sampler syringe using a sandwich technique with a 0.5 μL upper air gap was used to sample 3 μL of each pheromone extract and inject at a rate of 1.5 μL/sec into a Varian 3500 Gas Chromatograph (Agilent Technologies, Santa Clara, CA). The GC was equipped with a heated injector fitted with a 4 mm ID open-top glass uniliner (Restek Corp., Bellefonte, PA) containing glass wool, a fused silica capillary column 15 m × 0.25 mm with 0.25 μm Stabilwax^®^ (Restek Corp.) film thickness, a 5 m × 0.25 mm fused silica guard column, and a flame ionization detector. The gas chromatograph was programmed for a 20 min run time as follows: injector temperature 200°C, splitless for 1.5 min, then split for the remainder of the run (split ratio 50:1 at 60°C); detector temperature 250°C, attenuation set at 32^−11^; column oven programmed at 80°C held for 2 min, temperature ramp from 60 to 240°C at 10°/min, held at 240°C for 5 min to end of the run. Hydrogen was used as the carrier gas at a flow rate of 20 cm/sec (6.5 psi head pressure) and nitrogen was used as makeup gas. Under these conditions, the 7-TDA internal standard and the two pheromone isomers (*E*- and *Z*-11-tetradecenyl acetate) eluted at ≈ 13.1–13.5 min with each of the three peaks separated by 0.2 to 0.4 min. Chromatograms were used to estimate the ratio of *E*- to *Z*-11-14:OAc isomers by comparing the area under the isomers’ peaks at the appropriate retention times. Samples containing insufficient quantity of pheromone for detection, as indicated by the lack of peaks at the appropriate retention times, were not classified and were indicated as unresolved (U).

Each F_4_ family (1–6) was treated as an independent replicate, and a Pearson product-moment correlation coefficient (PMCC) was estimated between phenotype and genotype within and across families using SAS 9.2 (SAS Institute, Cary, NC). Female phenotype determined from pheromone gland component separation on the GC (≥90% *E*11-14:OAc isomer = 1.0, ≤5% *E*11-14:OAc isomer = 3.0, and all remaining estimated ratios = 2.0) was paired with corresponding *pgfar* genotypes from the same female (*pgfar-e*/*pgfar-e* = 0.0, *pgfar-e*/*pgfar-z* = 0.5, and *pgfar-z*/*pgfar-z* = 1.0). Strength of the linear relationship between the dependent (*pgfar* genotype) and independent variables (female GC isomer designation) were evaluated within each colony and across all intercrossed lines and pure *E*- and *Z*-race laboratory control colonies.

### Estimates of field hybridization among pheromone races

Adult *O. nubilalis* were collected using light traps at 16 locations from the Midwest and East Coast of the United States (Table 1; Fig. 2), where sites 1 to 5 were from geographic regions historically known to have pure *Z*-race populations. In contrast, collection sites 6 to 16 within the East Coast region of the United States were anticipated to have populations comprised of both *E*- and *Z*-race moths. The sex of each moth was determined visually, and DNA was extracted as described by Coates and Hellmich (71). Individuals were genotyped at the three *pgfar* SNP markers assayed with *Taq*I, *Nde*II, and *Mse*I as described above. Observed heterozygosity (*H*_O_) and expected heterozygosity (*H*_E_) and significance of the deviations from Hardy–Weinberg equilibrium (HWE) were tested for each *pgfar* SNP marker within all populations with Markov chain exact tests using the Arlequin 3.5.1.2 (Excoffier and Lischer 16). Differences between male and female *H*_O_ estimates were evaluated by Chi-square (χ^2^) tests using SAS 9.2 (SAS Institute). Hierarchical *F*-statistics and analysis of molecular variance (AMOVA) were also generated using Arlequin 3.5.1.2 (Excoffier and Lischer 16) after subdividing populations into Midwest (sites 1 to 5) and East Coast (sites 7 to 16) groupings. Significance was evaluated based on 1000 permutations as described by Weir and Cockerham (79). Pairwise *F*_ST_ estimates between sample sites were calculated by Arlequin 3.5.1.2 (Excoffier and Lischer 16), and significance thresholds set at a modified α according to the B–Y method (Benjamini and Yekutieli 68).

## Results

### Development of pheromone race-specific molecular genetic markers

**Figure 3:**
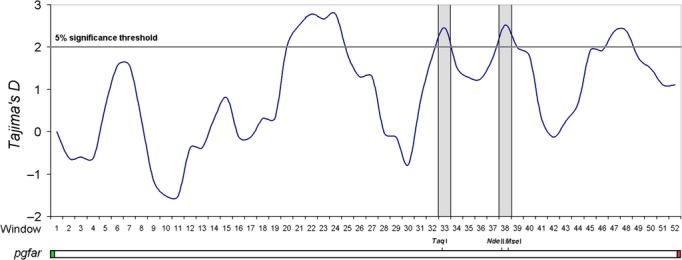
Estimates of directional selection using *Tajima's D* by analysis of 100-bp sliding windows with 25-bp iterations across the *Ostrinia nubilalis* pheromone gland fatty-acyl reductase (*pgfar*) gene. This figure was adapted from a similar analysis by Lassance et al. (35) to show the location single nucleotide polymorphisms (SNPs) detected by markers at *Taq*I, *Nde*II, and *Mse*I restriction endonuclease sites.

Alignment of cDNA sequences from the *O. nubilalis pgfar* gene produced a 1607-bp consensus sequence predicted to contain 1317-bp (439 aa) and 1326-bp (442 aa) coding regions for sequences derived from *E*- and *Z*-races, respectively (Fig. S1). Nucleotide diversity (*d*) (mean number of base substitutions per site) across all *pgfar* cDNAs was estimated as 0.013 ± 0.005. When partitioned within and between cDNAs derived from *Z*- and *E*- pheromone races, *d* was 0.002 ± 0.001 and 0.011 ± 0.005, respectively. The nucleotide diversity index (*π*) and *Tajima's D* estimate across the entire *pgfar* coding sequence was 0.020 and 1.614, respectively. Estimates of *Tajima's D* within 100-bp sliding windows across the *pgfar* coding sequence ranged from 1.514 to 2.778, and revealed four regions of significant sequence divergence between *Z*- and *E*-race *O. nubilalis* (significance threshold set at 2.0 as described for the same analysis performed by Lassance et al. 35; Fig. 3). Three putative *pgfar* allele-specific polymorphisms (SNPs) were identified in these regions of significant *Tajima's D* estimates that were also within restriction endonuclease (RE) sites (Fig. 3). Nucleotide positions G^857^, T^995^, and T^1005^ were fixed among cDNA sequences from *E*-race (*pgfar-e* alleles), and T^857^, G^995^, and G^1005^ nucleotides were fixed among cDNAs derived from *Z*-race (*pgfar-z* alleles). Nucleotides T^857^, T^995^, and T^1005^ complete palindromes for *Taq*I (TCGA), *Nde*II (GATC), and *Mse*I recognition sites (TTAA).

Oligonucleotide primers flanking the putative polymorphic *pgfar* RE sites were designed, and PCR-amplified fragments ranged from 145 to 150 bp. Restriction digest of the corresponding PCR-amplified products by *Taq*I, *Nde*II, and *Mse*I generated 118 + 32-bp, 83 + 62-bp, and 93 + 52-bp PCR-RFLP fragments, respectively (Fig. S2). The fragment sizes estimated from electrophoretic RFLP patterns were approximately the same as predicted from DNA sequence data for each allele (Table 2 footnotes). A pure *Z*-race population from Crawfordsville, IA (*n* = 48) and samples from the pure *E*-race BENY colony (*n* = 44) were genotyped at the *pgfar* loci to provide preliminary validation of the PCR-RFLP assay. The results for the Crawfordsville, IA *Z*-race samples (Table 1) indicated fixation of the nucleotides T^857^, G^995^, and G^1005^ predicted for *pgfar-z* alleles (Fig. S1). In contrast, PCR-RFLP results from the BENY colony (Table 1) were fixed for G^857^, T^995^, and T^1005^ as predicted for *pgfar-e* alleles. Thus, the Crawfordsville, IA and BENY samples were fixed for alternate *pgfar-z* and *pgfar-e* alleles (*H*_O_ = 0), and thus the genotypes were able to predict the phenotypes known to be fixed within both of these DNA sources (the pure *Z*-race population from Crawfordsville, IA was fixed for the *pgfar-z* allele, and the pure *E*-race BENY colony was fixed for the *pgfar-e* allele).

### Correlation between genotype and pheromone production phenotype

**Figure 4:**
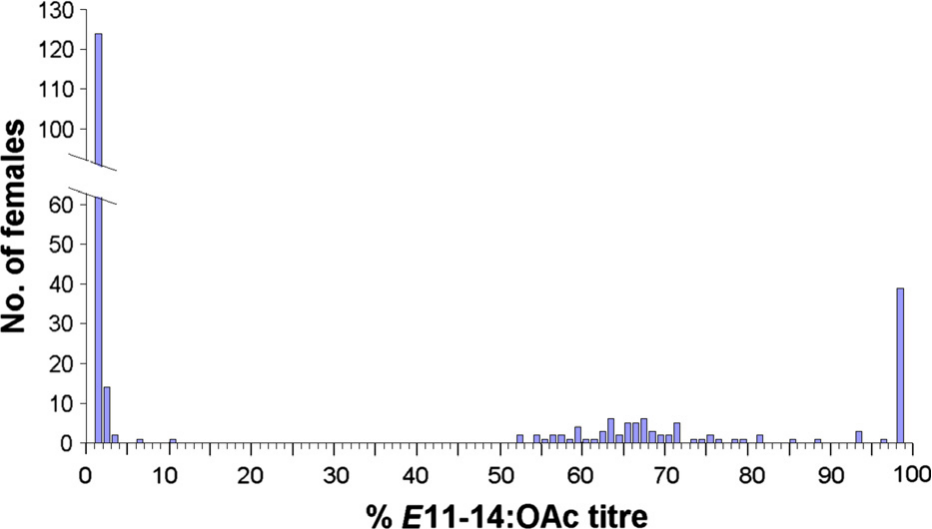
Histogram of *E*-Δ11-tetradecenyl acetate titers from GC analysis of F_4_ female *Ostrinia nubilalis* pheromone gland extracts presented as bulk analysis across six independent intercrossed families initiated between and *E*- and *Z*- race parents.

The correspondence between PCR-RFLP defined *pgfar-z* and *pgfar-e* alleles and female pheromone gland-produced *E*11- and *Z*11-14:OAc ratios were determined for segregating F_4_ females from replicate lines generated by mass mating of *E*- and *Z*-races in the parental generation. Pheromone glands were dissected from a total of 269 female *O. nubilalis* from Families 1 to 6, of which 247 provided GC readings from Families 1 (*n* = 45), 2 (*n* = 46), 3 (*n* = 46), 4 (*n* = 30), 5 (*n* = 26), and 6 (*n* = 21), as well as control colonies for *E*-race (*n* = 17) and *Z*-race (*n* = 16). Pheromone gland-derived hydrocarbons from 251 females were detected by GC (Table S1), where the proportion of the *E*11-14:OAc isomer in extracts ranged from 2 to 99 across all samples and all replicate lines (Fig. 4). A total of 259 female samples were genotyped using *Taq*I, *Nde*II, and *Mse*I PCR-RFLP assays, of which 244 samples had both genotypic and GC assay results. The proportions of *E*11-14:OAc among homozygous *pgfar-e*, heterozygous, and observed homozygous *pgfar-z* genotypes were 98.02 ± 4.24 (*n* = 42), 67.58 ± 8.92 (*n* = 59), and 2.74 ± 6.24 (*n* = 143), respectively. Among the seven female genotypes that showed potential discrepancies between SNP marker and GC data, five had *E*11-14:OAc ratios that ranged from 57 to 68 but were genotyped as homozygous *pgfar-z* at the *Taq*I-assayed SNP marker (T^857^/T^857^). The same five samples were genotyped as heterozygotes (*pgfar-e*/*pgfar-z*) for both *Nde*II- (G^995^/T^995^) and *Mse*I-assayed (G^1005^/T^1005^) SNP markers (Table S1).

**Figure 5:**
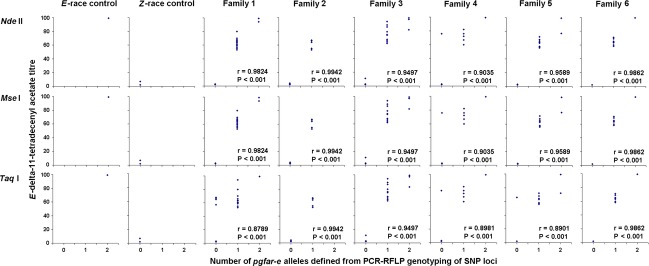
Scatter plots of female *Ostrinia nubilalis* pheromone gland *E*-Δ11-tetradecenyl acetate titers from GC analysis versus increasing number of pheromone gland-acyl reductase alleles from *E*-race (*pgfar-e*) identified from PCR-RFLP assays of three race-associated SNP loci (0 = *pgfar-z*/*pgfar-z*, 1 = heterozygous *pgfar-e*/*pgfar-z*, and 2 = *pgfar-e*/*pgfar-e* genotypes).

The Pearson product-moment correlation coefficient between the proportion of *E*11 and *Z*11-14:OAc pheromones produced by female *O. nubilalis* pheromone glands and the number of *pgfar-e* alleles from *Taq*I, *Nde*II, and *Mse*I SNP marker assays showed a positive relationship. Specifically, the correlation coefficient (*r*) ranged from 0.8789 to 0.9942 across all markers (*Taq*I, *Nde*II, and *Mse*I) and each replicated line (Family 1 to 6), and all were significant (*P*-values < 0.001; Fig. 5.). Mean correlation coefficients for data pooled across all lines were high for *Taq*I (*r* = 0.9699 ± 0.0321), *Nde*II (*r* = 0.9884 ±0.0182), and *Mse*I-assayed SNP markers (*r* = 0.9884 ±0.0182) (*P*-values < 0.001).

### Estimates of field hybridization among pheromone races

A total of 499 light trap-collected adult *O. nubilalis* were genotyped using the three *pgfar* SNP (PCR-RFLP) markers (Table 1; Fig. 2). The estimated mean *pgfar-e* allele frequencies at SNP loci G^857^, T^995^, and T^1005^ were 0.204 ± 0.100 (mean *H*_O_ of 0.107 ± 0.105; range 0.000–0.419) across all populations and across both sexes. Sample sites from historically *Z*-race regions of the Midwest United States (Lexington, KY; Crawfordsville, IA; Kanawha, IA; Mead, NE; and Brookings, SD) were fixed for SNPs T^857^, G^995^, and G^1005^, and were fixed for the *pgfar-z* allele (*H*_O_ = 0) as anticipated from historical data. In contrast, light trap samples from 11 East Coast locations (sites 6 through 16; Fig. 2) showed an estimated mean *H*_O_ across sites of 0.154 ± 0.110, and confirmed the prediction that heterogeneous *E*- and *Z*-race populations were present within this geographic region. SNP genotypes from 9 of 11 East coast locations did not significantly deviate from HWE across all SNP marker loci (*P* ≥ 0.082), with significant deviations shown among genotypes from Cohansey, NJ (*P*-value < 0.001), and Newark, DE (*P*-value < 0.001; remaining data not shown). When partitioned into male and female samples, the mean *H*_O_ did not deviate significantly between sexes at any location (*P*-values < 0.05; remaining data not shown). Global estimates of *F*-statistics indicated significant *pgfar* allele frequency variation among geographic regions (Midwest [sites 1 to 5] vs. East Coast [sites 7 to 16]; *F*_ST_ = 0.0782; *P* ≤ 0.001; Table S2). Pairwise *F*_ST_ estimates among light trap collection sites ranged from −0.02798 to 0.5156, and 17 of 55 comparisons (30.9%) were significant between Midwest and East Coast sample sites (*P*-values ≤ 0.009; B–Y significance threshold = 0.014) (Table S3). In contrast, seven of 65 pairwise *F*_ST_ estimates (10.8%) were significant among East Coast samples. Additionally, significant inbreeding was detected within Midwestern and within East Coast samples (F_IS_ = 0.3133; *P*-value < 0.001; Table S2). The significant variation between population from the Midwest compared with the East Coast were expected due to historical geographic range of *E*-race populations within East Coast regions. Thus, these results could be considered complementary to the laboratory genotype–phenotype association studies performed above.

## Discussion

### Development of pheromone race-specific molecular genetic markers

The two pheromone races of *O. nubilalis* show partial reproductive isolation when in sympatry, and may represent incipient species in the early stages of divergence (Dopman et al. 73; Lassance et al. 35). *E*- and *Z*-race individuals are morphologically indistinguishable, but females from each race can be identified by analysis of fatty acid derivatives produced in the pheromone gland (Klun and Brindley 30; Kochansky et al. 32; Roelofs et al. 77). Correspondence of phenotype with genotypes (mutations) has been established for insecticide resistance traits (Ffrench-Constant et al. 17), but population associations can be complicated by effects of inbreeding, population structure or selection (Berlocher and McPheron 4; Baxter et al. 3). The population association of the three molecular genetic markers developed in the current study was facilitated by prior knowledge that *O. nubilalis* pheromone production traits segregating in backcross pedigrees are linked to the *pgfar* locus (Lassance et al. 35). Despite this association between *pgfar* alleles and female pheromone within pedigrees, marker association within populations requires linkage disequilibrium (LD) in the face of continued genetic recombination (Jorde 26). The high levels of nucleotide diversity and lack of shared ancestral polymorphism between cDNAs derived from *E*- and *Z*-race *O. nubilalis*, along with significant *Tajima's D* estimates, suggested to Lassance et al. (35) that directional selection is acting on the *pgfar* locus.

The three SNP markers within *O. nubilalis pgfar* that were assayed by PCR-RFLP in this study are positioned in regions that were significantly affected by directional selection (based on sliding window estimates of *Tajima's D* < 2.0). Although this analysis was replicated from that previously performed by Lassance et al. (35), our similar analysis allowed positioning of the SNP markers within regions showing evidence of directional selection (Fig. 2). Furthermore, two of the three SNPs were at second codon positions and caused nonsynonymous (amino acid changing) mutations that were fixed differentially among *E*- and *Z*-race cDNAs. Specifically, the *Taq*I PCR-RFLP assay detected a G/T^857^ SNP that results in a deduced Cys to Phe change, and the *Nde*II PCR-RFLP assayed for a T/G^995^ SNP that predicts an Ile to Ser change. The T/G^1005^ SNP detected by the *Mse*I PCR-RFLP assay is at a 3rd codon position, but is associated with a nonsynonymous Val to Met change that is due to mutation of the 1st codon position (Val [GTT] to Met [ATG]). Initial population screening of known *E*- and *Z*-race samples, followed by more broad population sampling and association studies, indicated that the three SNP markers are diagnostic for the differentiation of *O. nubilalis* pheromone races collected in the field. Focusing on amino acid changing SNPs in population association studies has been proposed, because significant associations may have a higher probability of being detected at nonsynonymous sites (Botstein and Risch 6). The accumulation of mostly nonsynonymous changes within *O. nubilalis pgfar* suggests that divergent selection has resulted in enzymes with derived specificities for the production of *E*- and *Z*-stereoisomers.

### Correlation between genotype and pheromone production

SNP markers consist of base substitutions at a single genomic locus, where individual mutations are generally biallelic and have lower allele diversities and provide less statistical power to discriminate unique genotypes compared to microsatellite loci (Xing et al. 80). However, SNPs are increasingly being used for population genetics and mapping studies due to their abundance, and relative ease of discovery and development (Glaubitz et al. 21; Morin et al. 47). SNP markers were previously developed from *O. nubilalis* expressed sequence tags (ESTs; Coates et al. 72, 10), and subsequently applied to detect significant levels of genetic differentiation among *O. nubilalis* populations (Coates et al. 10) and to determine linkage groups associated with *Bacillus thuringiensis* resistance traits (Coates et al. 11). SNPs have been used in other species of Lepidoptera to identify population genetic structure (Margam et al. 42) and to detect genome regions that influence traits (Sreekumar et al. 59). Our data show that the segregation of three SNP markers within the *O. nubilalis pgfar* gene are significantly correlated with the proportion of *E*11-14:OAc produced in the female pheromone gland. These results are consistent with previous analyses of backcross pedigree data (Lassance et al. 35). This evidence also indicates that linkage disequilibrium in this genome region between *O. nubilalis* pheromone races may be maintained by disruptive selection. The size of the *O. nubilalis* genome region affected by LD remains unknown, but within other species where the level of LD is known haplotype blocks have been shown to span regions of ~500 kb (Hirschhorn and Daly 24). Fourteen putative loci were identified and proposed to be involved in adaptive divergence between the species *O. nubilalis* and *O. scapulalis* (Midamegbe et al. 46), but analogous genome wide studies have not been reported between *E*- and *Z*-race *O. nubilalis*.

Despite the strong correlation between *pgfar* genotype and female phenotype, estimated ratios of *E*11-14:OAc in F_4_ females with *pgfar-e*/*pgfar-z* genotypes ranged from 53 to 94 (mean 67.3 ± 9.0), and often deviated from the expected 65:35 *E*11- to *Z*11-14:OAc hybrid ratio (Klun and Maini 31; Roelofs et al. 52). Reciprocal crosses between *E*- and *Z*-race *O. nubilalis* in previous studies indicated that modifier loci may affect female pheromone production, causing shifts in heterozygote pheromone blend ratios, and were most noticeable in female F_1_ backcrosses to the *Z*-race (Löfstedt et al. 39; Zhu et al. 67). The effect of segregating modifier loci on female pheromone blend ratios may be responsible for the skewed ratios we observed among heterozygous F_4_ females, but additional experiments will be required to determine the genetic loci involved. The rate of mating success for F_1_ hybrid females from United States population that express intermediate pheromone blend ratios showed no significant difference in backcrosses to either *E*- or *Z*-race males in laboratory experiments (Pelozuelo et al. 50), and suggest that these backcross individuals may also be present in natural populations.

### Estimates of field hybridization among pheromone races

Mate attraction in *O. nubilalis* may be a function of independent genetic loci; the *Pher* (=*pgfar*) locus that determines female pheromone production, and *Resp* and *Olf* loci that influence male behavioral response and structure of the male antennae, respectively (Hansson et al. 23; Roelofs et al. 53; Dopman et al. 12). The accumulation of genetic and phenotypic differences between *E*- and *Z*-race adults may lead to preferential mating within each race. Regardless, hybridization occurs in both the field and laboratory, where pairings of *E*-race male with *Z*-race females tend to be more frequent than the reciprocal cross (Liebherr and Roelofs 36; Linn et al. 37; Pelozuelo et al. 50). Male attraction to females of the opposite pheromone race decreases significantly with increasing distances (Dopman et al. 13), and successful mating may also depend upon recognition of a male courtship pheromone at close range as well as courtship behaviors and signaling (Lassance and Löfstedt 34; Takanashi et al. 60). Despite chemical and behavioral mechanisms of prereproductive isolation between *E*- and *Z*-races, accurate estimation of the strength of this reproductive barrier in natural populations has been elusive (see Introduction).

Limited gene flow has been detected between *O. nubilalis* pheromone races using anonymous genetic markers (Harrison and Vawter 75; Cardè et al. 69; Cianchi et al. 70; Glover et al. 22). Willett and Harrison (65) concluded from analysis of variation in the pheromone binding protein gene that gene flow occurs between the *E*- and *Z*-races in New York. Hybrid female frequencies of 5%, 11%, and 12% were estimated at three locations in North Carolina (Durant et al. 15), and 0–22% was observed among sites in New York (Roelofs et al. 52), but estimates were based on GC analysis of a limited number of pheromone glands. Up to this point, estimation of hybrid formation within field populations has remained an impasse for population genetic studies due to difficulties in performing GC analysis upon dissected pheromone glands of wild caught virgin females. The novelty and utility of the current study is that molecular genetic markers developed herein discriminate *pgfar-e* and *pgfar-z* alleles within field populations, and for the first time offer a relatively high throughput method for identifying phenotypes for population genetic studies. The markers were applied to detect *pgfar-e*/*pgfar-z* heterozygotes, and subsequently to estimate hybridization frequency between *E*- and *Z*-race *O. nubilalis*. Genotyped individuals (*n* = 331) from light trap samples collected in regions of the United States with sympatric *E*- and *Z*-race populations indicated hybridization frequencies ranged from 0 to 41.9%. The assortative mating model assumes that attraction between *E*- and *Z*-race *O. nubilalis* is rare (Roelofs et al. 53), but our genotype data suggest that intermating of *E*- and *Z*-races can occur at relatively high frequencies in natural populations in the Eastern United States. Given that mate attraction among pheromone races is weak at long distances (Dopman et al. 13), the breakdown in assortative mating in the field may take place at close distances. Adults tend to concentrate in grassy “aggregation sites” during the night for mating (Showers et al. 56, 57), which perhaps would promote close-range encounters between *E*- and *Z*-race moths if temporal mating periods overlap.

Given the observed moderate to high levels of hybridization, assortative mating may not be the only factor reinforcing the genetic isolation between *E*- and *Z*-races. Females with 70, 72, and 73% *E*11-14:OAc were previously detected in New York, and may be evidence that backcross females are produced in natural populations (Roelofs et al. 52), but genetic tests for backcross females remain undeveloped pending the identification of modifier loci (Löfstedt et al. 39; Zhu et al. 67). Furthermore, the reproductive fate of hybrid males remains obscure. Glover et al. (22) showed that most heterozygous (F_1_ hybrid) males derived from reciprocal cross do not respond to pheromone blends produced by either *E*- or *Z*- race females. The infrequent hybrids that did respond showed no pheromone blend preference. Thus, F_1_ hybrid males might be a genetic dead end that are incapable of mating (Lassance 33), and might impose a partial barrier to the introgression of genes between pheromone races. The development and application of markers for the male *Resp* locus (Dopman et al. 74) as well as genes involved in the modification of female pheromone blend ratios among backcrosses in *O. nubilalis* (Löfstedt et al. 39; Zhu et al. 67) will likely be important in understanding direction of gene flow and the reproductive fate of hybrid within natural populations.

## Supporting Information

**Table S1**. The *pgfar* genotypes and corresponding gas chromatograph (GC)-determined proportion of *E*11-14:OAc among females from Families 1–6

**Table S2**. Results of hierarchical population genetic structure using analysis of molecular variance (AMOVA.).

## References

[b1] Anglade P, Stockel J (1984). Intraspecific sex-pheromone variability in the European corn borer, *Ostrinia nubilalis* Hbn (Lepidoptera, Pyralidae). Agronomie.

[b2] Bartels DW, Hutchison WD, Udayagiri S (1997). Pheromone trap moitoring of *Z*-strain European corn borer (Lepidoptera: Pyralidae): optimum pheromone blend, comparison with black light trap, and trap number requirements. J. Econ. Entomol.

[b3] Baxter SW, Madeau NJ, Maroja LS, Wilkinson P, Counterman BA, Dawson A (2010). Genome hotspot for adaption: the population genetics of Mullerian mimicry in the Heliconius melpomene clade. PLoS Genet.

[b68] Benjamini Y, Yekutieli D (2001). The control of false discovery rate under dependency. Ann. Stat.

[b4] Berlocher SH, McPheron BA (1996). Population structure of Rhagoletis pomonella, the apple maggot fly. Heredity.

[b6] Botstein D, Risch N (2003). Discovering genotypes underlying human phenotypes: past successes for mendelian disease, future approaches for complex disease. Nat. Genet.

[b69] Cardé RT, Roelofs WL, Harrison RG, Vawter AT, Brussard PF, Mutuura A (1978). European corn borer: pheromone polymorphism or sibling species?. Science.

[b70] Cianchi R, Maini R, Bullini L (1980). Genetic distance between pheromone strains of the European corn borer *Ostrinia nubilalis* different contribution of variable substrate regulatory, and nonregulatory enzymes. Heredity.

[b71] Coates BS, Hellmich RL (2003). Two sex-chromosome-linked microsatellite loci show geographic variance among North American *Ostrinia nubilalis*. J. Insect Sci.

[b72] Coates BS, Sumerford DV, Hellmich RL, Lewis LC (2008). Mining an *Ostrinia nubilalis* midgut expressed sequence tag (EST) library for candidate genes and single nucleotide polymorphisms (SNPs). Insect Mol. Biol.

[b10] Coates BS, Bayles DO, Wanner KW, Robertson HM, Hellmich RL, Sappington TW (2011a). The application and performance of single nucleotide polymorphism (SNP) markers for population genetic analyses of Lepidoptera. Front. Genet.

[b11] Coates BS, Sumerford DV, Lopez MD, Wang H, Fraser LM, Kroemer JA (2011b). A single major QTL controls the expression of a larval Cry1F resistance trait in *Ostrinia nubilalis* (Lepidoptera: Crambidae). Genetica.

[b12] Dopman EB, Bognanowicz SM, Harrison RG (2004). Genetic mapping of sexual isolation between E and Z pheromone strains of the European corn borer (*Ostrinia nubilalis*. Genetics.

[b13] Dopman EB, Robbins PS, Seaman A (2009). Components of reproductive isolation between North American pheromone strains of the European corn borer. Evolution.

[b73] Dopman EB, Robbins PS, Seaman A (2010). Components of reproductive isolation between North American pheromone strains of the European corn borer. Evolution.

[b74] Dopman EB, Pérez L, Bogdanowicz SM, Harrison HG (2005). Consequences of reproductive barriers for genealogical discordance in the European corn borer. Proc. Natl. Acad. Sci. USA.

[b15] Durant JA, Fescemyer HW, Mason CE, Udayagiri S (1995). Effectiveness of four blends of European corn borer (Lepidoptera: Pyralidae) sex pheromone isomers at three locations in South Carolina. J. Agric. Entomol.

[b16] Excoffier L, Lischer HE (2010). Arlequin suite ver 3.5: a new series of programs to perform population genetics analyses under Linux and Windows. Mol. Ecol. Resour.

[b17] Ffrench-Constant RH, Rocheleau TA, Steichen JC, Chalmers AE (1993). A point mutation in a Drosophila GABA receptor confers insecticide resistance. Nature.

[b20] Gentile G, Della TA, Maegga B, Powell JR, Caccone A (2002). Genetic differentiation in the African malaria vector, Anopheles gambiae s.s., and the problem of taxonomic status. Genetics.

[b21] Glaubitz JC, Rhodes OE, Dewoody JA (2003). Prospects for inferring pairwise relationships with single nucleotide polymorphism. Mol. Ecol.

[b22] Glover TJ, Campbell MG, Linn CE, Roelofs WL (1991). Unique sex chromosome mediated behavioural response specificity of hybrid male European corn borer moths. Experientia.

[b75] Harrison RG, Vawter AT (1977). Allozyme differentiation between pheromone strains of the European corn borer, *Ostrinia nubilalis*. Ann. Entomol. Soc. Am.

[b23] Hansson B, Lofstedt C, Roelofs W (1987). Inheritance of olfactory response to sex pheromone components in *Ostrinia nubilalis*. Naturwissenschaften.

[b24] Hirschhorn JN, Daly MJ (2005). Genome-wide association studies for common diseases and complex traits. Nat. Rev. Genet.

[b26] Jorde LB (2000). Linkage disequilibrium and the search for complex disease genes. Genome Res.

[b29] Klun JA, Cooperators (1975). Insect sex pheromones: intraspecific pheromonal variability of *Ostrinia nubilalis* in North America and Europe. Environ. Entomol.

[b30] Klun JA, Brindley TA (1970). cis-11-tetradecenyl acetate, a sex stimulant of the European corn borer. J. Econ. Entomol.

[b31] Klun JA, Maini S (1979). Genetic basis of an insect chemical communication system: the European corn borer. Environ. Entomol.

[b32] Kochansky J, Carde RT, Liedherr J, Roelofs WL (1975). Sex pheromone of the European corn borer, *Ostrinia nubilalis* (Lepidoptera: Pyralidae), in New York. J. Chem. Ecol.

[b33] Lassance JM (2010). Journey in the Ostrinia world: from pest to model in chemical ecology. J. Chem. Ecol.

[b34] Lassance JM, Löfstedt C (2009). Concerted evolution of male and female display traits in the European corn borer, *Ostrinia nubilalis*. BMC Biol.

[b35] Lassance JM, Groot AT, Lienard MA, Antony B, Borgwardt C, Andersson F (2010). Allelic variation in a fatty-acyl reductase gene causes divergence in moth sex pheromones. Nature.

[b36] Liebherr J, Roelofs W (1975). Laboratory hybridization and mating period studies using 2 pheromone strains of *Ostrinia nubilalis*. Ann. Entomol. Soc. Am.

[b37] Linn C, Young M, Gendle M, Glover T, Roelofs W (1997). Sex pheromone blend discrimination in two races and hybrids of the European Corn Borer moth, *Ostrinia nubilalis*. Physiol. Entomol.

[b39] Löfstedt C, Hansson BS, Roelofs W, Bengtsson BO (1989). No linkage between genes controlling female pheromone production and male pheromone response in the European corn borer, *Ostrinia nubilalis* Hubner (Lepidoptera; Pyralidae). Genetics.

[b40] Ma PWK, Roelofs WL (2002). Sex pheromone gland of the female European corn borer moth, *Ostrinia nubilalis* (Lepidoptera, Pyralidae): ultrastructural and biochemical evidences. Zoolog. Sci.

[b41] Machado CA, Kliman RM, Markert JA, Hey J (2002). Inferring the history of speciation from multilocus DNA sequence data: the case of Drosophila pseudoobscura and close relatives. Mol. Biol. Evol.

[b42] Margam VM, Coates BS, Bayles DO, Hellmich RL, Agunbiade T, Seufferheld MJ (2011). Transcriptome sequencing, and rapid development and application of SNP markers for the legume pod borer Maruca vitrata (Lepidoptera: Crambidae). PLoS ONE.

[b44] Mason CE, Ehresman NP, He K, Italia AS, Pesek JD (1997). Performance of three commercial pheromone sources for trapping European corn borer. Proceeding from the 31st Northeast Regional Field Crops Insect Conference at Burlington.

[b46] Midamegbe A, Vitalis R, Malausa T, Delava E, Cros-Arteil S, Streiff R (2011). Scanning the European corn borer (Ostrinia spp.) genome for adaptive divergence between host-affiliated sibling species. Mol. Ecol.

[b47] Morin PA, Luikart G, Wayne RK, Grp SW (2004). SNPs in ecology, evolution and conservation. Trends Ecol. Evol.

[b49] O'Rourke ME, Sappington TW, Fleischer SJ (2010). Managing resistance to Bt crops in a genetically variable insect herbivore, *Ostrinia nubilalis*. Ecol. Appl.

[b76] Pelozuelo L, Frerot B (2008). Monitoring of the European corn borer moth, *Ostrinia nubilalis* Hub., with pheromone baited traps: review of trapping system basics and remaining problems. J. Econ. Entomol.

[b50] Pelozuelo L, Muesnier S, Audiot P, Bourguet D, Ponsard S (2007). Assortative mating between European corn borer pheromone races: Beyond assortative mating. PLoS ONE.

[b51] Roelofs WL, Rooney AP (2003). Molecular genetics and evolution of pheromone biosynthesis in Lepidoptera. Proc. Natl Acad. Sci. USA.

[b77] Roelofs WL, Cardé RT, Bartell RJ, Tierney PG (1972). Sex attractant trapping of the European corn borer in New York. Environ. Entomol.

[b52] Roelofs WL, Du JW, Tang XH, Robbins PS, Eckenrode CJ (1985). Three European corn borer populations in New York based on sex pheromones and voltinism. J. Chem. Ecol.

[b53] Roelofs WL, Glover TJ, Tang XH, Sreng I, Robbins P, Eckenrode C (1987). Sex pheromone production and perception in European corn borer moth is determined by both autosomal and sex-linked genes. Proc. Natl Acad. Sci. USA.

[b54] Roelofs WL, Liu W, Hao G, Jiao H, Rooney AP, Linn CE (2002). Evolution of moth sex pheromones via ancestral genes. Proc. Natl Acad. Sci. USA.

[b55] Schulze TG, McMahon FJ (2004). Defining the phenotype in human genetic studies: forward genetics and reverse phenotyping. Hum. Hered.

[b56] Showers WB, Reed GL, Oloumi-Sadeghi H (1974). Mating studies of female European corn borers: relationship between deposition of egg masses on corn and captures in light traps. J. Econ. Entomol.

[b57] Showers WB, Reed GL, Robinson JF, Derozari MB (1976). Flight and sexual activity of the European corn borer. Environ. Entomol.

[b58] Smith FS, Pierce HD, Borden JH (1991). Sex pheromone of the mullein bug, Campylomma verbasci (Meyer) (Heteroptera: Miridae). J. Chem. Ecol.

[b59] Sreekumar S, Aswath SK, Slathia M, Kumar SN, Qadri SMH (2011). Detection of a single nucleotide polymorphism (SNP) DNA marker linked to cocoon traits in the mulberry silkworm, Bombyx mori (Lepidoptera: Bombycidae). Eur. J. Entomol.

[b60] Takanashi T, Nakano R, Surlykke A, Tatsuta H, Tabata J, Ishikawa Y (2010). Variation in courtship ultrasounds of three Ostrinia moths with different sex pheromones. PLoS ONE.

[b61] Tamura K, Dudley J, Nei M, Kumar S (2007). MEGA4: Molecular Evolutionary Genetics Analysis (MEGA) software version 4.0. Mol. Biol. Evol.

[b62] Tsuchihashi Z, Dracopoli NC (2002). Progress in high throughput SNP genotyping methods. Pharmacogenomics J.

[b63] Untergasser A, Nijveen H, Rao X, Bisseling T, Geurts R, Leunissen JAM (2007). Primer3Plus, an enhanced web interface to Primer3. Nucleic Acids Res.

[b78] Vignal A, Milan D, San Cristobal M, Eggen A (2003). A review on SNP and other types of molecular markers and their use in animal genetics. Genet. Sel. Evol.

[b64] Vinal SC (1917). The European corn borer *Pyrausta nubilalis* (Hübner), a recently established pest in Massachusetts. Mass. Agric. Exp. Stat. Bull.

[b79] Weir BS, Cockerham CC (1984). Estimating F-statistics for the analysis of population structure. Evolution.

[b65] Willett CS, Harrison RG (1999). Insights into genome differentiation: pheromone-binding protein variation and population history in the European corn borer (*Ostrinia nubilalis*. Genetics.

[b66] Wu CI (2001). The genic view of the process of speciation. J. Evol. Biol.

[b80] Xing C, Schumacher FR, Xing G, Lu Q, Wang T, Elston RC (2005). Comparison of microsatellites, single-nucleotide polymorphisms (SNPs) and composite markers derived from SNPs in linkage analysis. BMC Genet.

[b67] Zhu J, Löfstedt C, Bengtsson BO (1996). Genetic variation in the strongly canalized sex pheromone communication system of the European corn borer, *Ostrinia nubilalis* Hübner (Lepidoptera; Pyralidae). Genetics.

