# Supplementary material for: Frequency of hybridization between Ostrinia nubilalis E-and Z-pheromone races in regions of sympatry within the United States
Source: Ecol Evol. 2013 Jun 24;3(8):2459–70. doi: 10.1002/ece3.639 (PMC3930039; doi:10.1002/ece3.639)
Supplement: Supplementary file 1 [file ece30003-2459-SD1.doc]

**Figure. S1.** Multiple sequence alignment of *Ostrinia nubilalis* pheromone gland fatty-acyl reductase (*pgfar*) complementary DNA (cDNA) from females that produce *E*-Δ11-tetradecenyl acetate (*E*-race; black) and *Z*-Δ11-tetradecenyl acetate (*Z*-race; blue) as their major pheromone component. The GenBank accessions FU808256.1 to GU808276.1 are submissions made by Lassance et al. (2010). Shared ancestral single nucleotide polymorphism (SNPs; substitution mutations) that are shared between cDNAs from *E*- and *Z*-race are highlighted in light blue, unique derived SNPs with *E*- or *Z*-race are highlighted in yellow, and the unique derived SNPs that have reached fixation within *E*-race are highlighted in green. Corresponding amino acid translations are indicated above cDNAs.

M S A N T M E T D E Q F T

GU808256.1 CAGTCTAGAAAAATGTCAGCAAATACCATGGAAACTGATGAACAATTTAC 50

GU808257.1 CAGTCTAGAAAAATGTCAGCAAATACCATGGAAACTGATGAACAATTTAC 50

GU808258.1 CAGTCTAGAAAAATGTCAGCAAATACCATGGAAACTGATGAACAATTTAC 50

GU808259.1 CAGTCTAGAAAAATGTCAGCAAATACCATGGAAACTGATGAACAATTTAC 50

GU808260.1 CAGTCTAGAAAAATGTCAGCAAATACCATGGAAACTGATGAACAATTTAC 50

GU808261.1 CAGTCTAGAAAAATGTCAGCAAATACCATGGAAACTGATGAACAATTTAC 50

GU808262.1 CAGTCTAGAAAAATGTCAGCAAATACCATGGAAACTGATGAACAATTTAC 50

GU808263.1 CAGTCTAGAAAAATGTCAGCAAATACCATGGAAACTGATGAACAATTTAC 50

GU808264.1 CAGTCTAGAAAAATGTCAGCAAATACCATGGAAACTGATGAACAATTTAC 50

GU808265.1 CAGTCTAGAAAAATGTCAGCAAATACCATGGAAACTGATGAACAATTTAC 50

M S A N T M E T D E Q F T

GU808266.1 CAGTCTAGAAAAATGTCAGCAAATACCATGGAAACTGATGAACAATTTAC 50

GU808267.1 CAGTCTAGAAAAATGTCAGCAAATACCATGGAAACTGATGAACAATTTAC 50

GU808268.1 CAGTCTAGAAAAATGTCAGCAAATACCATGGAAACTGATGAACAATTTAC 50

GU808269.1 CAGTCTAGAAAAATGTCAGCAAATACCATGGAAACTGATGAACAATTTAC 50

GU808270.1 CAGTCTAGAAAAATGTCAGCAAATACCATGGAAACTGATGAACAATTTAC 50

GU808271.1 CAGTCTAGAAAAATGTCAGCAAATACCATGGAAACTGATGAACAATTTAC 50

GU808272.1 CAGTCTAGAAAAATGTCAGCAAATACCATGGAAACTGATGAACAATTTAC 50

GU808273.1 CAGTCTAGAAAAATGTCAGCAAATACCATGGAAACTGATGAACAATTTAC 50

GU808274.1 CAGTCTAGAAAAATGTCAGCAAATACCATGGAAACTGATGAACAATTTAC 50

GU808275.1 CAGTCTAGAAAAATGTCAGCAAATACCATGGAAACTGATGAACAATTTAC 50

GU808276.1 CAGTCTAGAAAAATGTCAGCAAATACCATGGAAACTGATGAACAATTTAC 50

Y N S P I V N F Y S G K S V F V T

GU808256.1 TTATAATTCACCAATTGTGAATTTTTACTCTGGAAAATCTGTTTTTGTTA 100

GU808257.1 TTATAATTCACCAATTGTGAATTTTTACTCTGGAAAATCTGTTTTTGTTA 100

GU808258.1 TTATAATTCACCAATTGTGAATTTTTACTCTGGAAAATCTGTTTTTGTTA 100

GU808259.1 TTATAATTCACCAATTGTGAATTTTTACTCTGGAAAATCTGTTTTTGTTA 100

GU808260.1 TTATAATTCACCAATTGTGAATTTTTACTCTGGAAAATCTGTTTTTGTTA 100

GU808261.1 TTATAATTCACCAATTGTGAATTTTTACTCTGGAAAATCTGTTTTTGTTA 100

GU808262.1 TTATAATTCACCAATTGTGAATTTTTACTCTGGAAAATCTGTTTTTGTTA 100

GU808263.1 TTATAATTCACCAATTGTGAATTTTTACTCTGGAAAATCTGTTTTTGTTA 100

GU808264.1 TTATAATTCACCAATTGTGAATTTTTACTCTGGAAAATCTGTTTTTGTTA 100

GU808265.1 TTATAATTCACCAATTGTGAATTTTTACTCTGGAAAATCTGTTTTTGTTA 100

Y N S P I V N F Y S G K S V F V T

GU808266.1 TTATAATTCACCAATTGTGAATTTTTACTCTGGAAAATCTGTTTTTGTTA 100

GU808267.1 TTATAATTCACCAATTGTGAATTTTTACTCTGGAAAATCTGTTTTTGTTA 100

GU808268.1 TTATAATTCACCAATTGTGAATTTTTACTCTGGAAAATCTGTTTTTGTTA 100

GU808269.1 TTATAATTCACCAATTGTGAATTTTTACTCTGGAAAATCTGTTTTTGTTA 100

GU808270.1 TTATAATTCACCAATTGTGAATTTTTACTCTGGAAAATCTGTTTTTGTTA 100

GU808271.1 TTATAATTCACCAATTGTGAATTTTTACTCTGGAAAATCTGTTTTTGTTA 100

GU808272.1 TTATAATTCACCAATTGTGAATTTTTACTCTGGAAAATCTGTTTTTGTTA 100

GU808273.1 TTATAATTCACCAATTGTGAATTTTTACTCTGGAAAATCTGTTTTTGTTA 100

GU808274.1 TTATAATTCACCAATTGTGAATTTTTACTCTGGAAAATCTGTTTTTGTTA 100

GU808275.1 TTATAATTCACCAATTGTGAATTTTTACTCTGGAAAATCTGTTTTTGTTA 100

GU808276.1 TTATAATTCACCAATTGTGAATTTTTACTCTGGAAAATCTGTTTTTGTTA 100

G A T G F L G T V L V E K L L F

GU808256.1 CTGGAGCTACAGGATTTCTGGGGACGGTTTTAGTTGAGAAACTGCTGTTC 150

GU808257.1 CTGGAGCTACAGGATTTCTGGGGACGGTTTTAGTCGAGAAACTGCTGTTC 150

GU808258.1 CTGGAGCTACAGGATTTCTGGGGACGGTTTTAGTCGAGAAACTGCTGTTC 150

GU808259.1 CTGGAGCTACAGGATTTCTGGGGACGGTTTTAGTCGAGAAACTGCTGTTC 150

GU808260.1 CTGGAGCTACAGGATTTCTGGGGACGGTTTTAGTTGAGAAACTGCTGTTC 150

GU808261.1 CTGGAGCTACAGGATTTCTGGGGACGGTTTTAGTCGAGAAACTGCTGTTC 150

GU808262.1 CTGGAGCTACAGGATTTCTGGGGACGGTTTTAGTCGAGAAACTGCTGTTC 150

GU808263.1 CTGGAGCTACAGGATTTCTGGGGACGGTTTTAGTCGAGAAACTGCTGTTC 150

GU808264.1 CTGGAGCTACAGGATTTCTGGGGACGGTTTTAGTCGAGAAACTGCTGTTC 150

GU808265.1 CTGGAGCTACAGGATTTCTGGGGACGGTTTTAGTCGAGAAACTGCTGTTC 150

G A T G F L G T V L V E K L L F

GU808266.1 CTGGAGCTACAGGATTTCTGGGGACGGTTTTAGTCGAGAAACTGCTGTTC 150

GU808267.1 CTGGAGCTACAGGATTTCTGGGGACGGTTTTAGTCGAGAAACTGCTGTTC 150

GU808268.1 CTGGAGCTACAGGATTTCTGGGGACGGTTTTAGTCGAGAAACTGCTGTTC 150

GU808269.1 CTGGAGCTACAGGATTTCTGGGGACGGTTTTAGTCGAGAAACTGCTGTTC 150

GU808270.1 CTGGAGCTACAGGATTTCTGGGGACGGTTTTAGTCGAGAAACTGCTGTTC 150

GU808271.1 CTGGAGCTACAGGATTTCTGGGGACGGTTTTAGTCGAGAAACTGCTGTTC 150

GU808272.1 CTGGAGCTACAGGATTTCTGGGGACGGTTTTAGTCGAGAAACTGCTGTTC 150

GU808273.1 CTGGAGCTACAGGATTTCTGGGGACGGTTTTAGTCGAGAAACTGCTGTTC 150

GU808274.1 CTGGAGCTACAGGATTTCTGGGGACGGTTTTAGTCGAGAAACTGCTGTTC 150

GU808275.1 CTGGAGCTACAGGATTTCTGGGGACGGTTTTAGTCGAGAAACTGCTGTTC 150

GU808276.1 CTGGAGCTACAGGATTTCTGGGGACGGTTTTAGTCGAGAAACTGCTGTTC 150

S C K G I N N I Y I L I K Q T E D

GU808256.1 TCTTGCAAAGGAATAAATAATATTTACATTTTGATAAAGCAGACAGAAGA 200

GU808257.1 TCTTGCAAAGGAATAAATAATATTTACATTTTGATAAAGCAGACAGAAGA 200

GU808258.1 TCTTGCAAAGGAATAAATAATATTTACATTTTGATAAAGCAGACAGAAGA 200

GU808259.1 TCTTGCAAAGGAATAAATAATATTTACATTTTGATAAAGCAGACAGAAGA 200

GU808260.1 TCTTGCAAAGGAATAAATAATATTTACATTTTGATAAAGCAGACAGAAGA 200

GU808261.1 TCTTGCAAAGGAATAAATAATATTTACATTTTGATAAAGCAGACAGAAGA 200

GU808262.1 TCTTGCAAAGGAATAAATAATATTTACATTTTGATAAAGCAGACAGAAGA 200

GU808263.1 TCTTGCAAAGGAATAAATAATATTTACATTTTGATAAAGCAGACAGAAGA 200

GU808264.1 TCTTGCAAAGGAATAAATAATATTTACATTTTGATAAAGCAGACAGAAGA 200

GU808265.1 TCTTGCAAAGGAATAAATAATATTTACATTTTGATAAAGCAGACAGAAGA 200

S C K G I N N I Y I L I K Q T E D

GU808266.1 TCTTGCAAAGGAATAAATAATATTTACATTTTGATAAAGCAGACAGAAGA 200

GU808267.1 TCTTGCAAAGGAATAAATAATATTTACATTTTGATAAAGCAGACAGAAGA 200

GU808268.1 TCTTGCAAAGGAATAAATAATATTTACATTTTGATAAAGCAGACAGAAGA 200

GU808269.1 TCTTGCAAAGGAATAAATAATATTTACATTTTGATAAAGCAGACAGAAGA 200

GU808270.1 TCTTGCAAAGGAATAAATAATATTTACATTTTGATAAAGCAGACAGAAGA 200

GU808271.1 TCTTGCAAAGGAATAAATAATATTTACATTTTGATAAAGCAGACAGAAGA 200

GU808272.1 TCTTGCAAAGGAATAAATAATATTTACATTTTGATAAAGCAGACAGAAGA 200

GU808273.1 TCTTGCAAAGGAATAAATAATATTTACATTTTGATAAAGCAGACAGAAGA 200

GU808274.1 TCTTGCAAAGGAATAAATAATATTTACATTTTGATAAAGCAGACAGAAGA 200

GU808275.1 TCTTGCAAAGGAATAAATAATATTTACATTTTGATAAAGCAGACAGAAGA 200

GU808276.1 TCTTGCAAAGGAATAAATAATATTTACATTTTGATAAAGCAGACAGAAGA 200

L T I E A R I L N Y L N S K A F H

GU808256.1 CCTGACCATTGAAGCGAGGATTTTAAATTATTTGAATTCGAAGGCTTTTC 250

GU808257.1 CCTGACCATTGAAGCGAGGATTTTAAATTATTTGAATTCGAAGGCTTTTC 250

GU808258.1 CCTGACCATTGAAGCGAGGATTTTAAATTATTTGAATTCGAAGGCTTTTC 250

GU808259.1 CCTGACCATTGAAGCGAGGATTTTAAATTATTTGAATTCGAAGGCTTTTC 250

GU808260.1 CCTGACCATTGAAGCGAGGATTTTAAATTATTTGAATTCGAAGGCTTTTC 250

GU808261.1 CCTGACCATTGAAGCGAGGATTTTAAATTATTTGAATTCGAAGGCTTTTC 250

GU808262.1 CCTGACCATTGAAGCGAGGATTTTAAATTATTTGAATTCGAAGGCTTTTC 250

GU808263.1 CCTGACCATTGAAGCGAGGATTTTAAATTATTTGAATTCGAAGGCTTTTC 250

GU808264.1 CCTGACCATTGAAGCGAGGATTTTAAATTATTTGAATTCGAAGGCTTTTC 250

GU808265.1 CCTGACCATTGAAGCGAGGATTTTAAATTATTTGAATTCGAAGGCTTTTC 250

L T I E A R I L N Y L N S K A F H

GU808266.1 CCTGACGATTGAAGCGAGGATTTTAAATTATTTGAATTCGAAGGCTTTTC 250

GU808267.1 CCTGACGATTGAAGCGAGGATTTTAAATTATTTGAATTCGAAGGCTTTTC 250

GU808268.1 CCTGACGATTGAAGCGAGGATTTTAAATTATTTGAATTCGAAGGCTTTTC 250

GU808269.1 CCTGACGATTGAAGCGAGGATTTTAAATTATTTGAATTCGAAGGCTTTTC 250

GU808270.1 CCTGACGATTGAAGCGAGGATTTTAAATTATTTGAATTCGAAGGCTTTTC 250

GU808271.1 CCTGACGATTGAAGCGAGGATTTTAAATTATTTGAATTCGAAGGCTTTTC 250

GU808272.1 CCTGACGATTGAAGCGAGGATTTTAAATTATTTGAATTCGAAGGCTTTTC 250

GU808273.1 CCTGACGATTGAAGCGAGGATTTTAAATTATTTGAATTCGAAGGCTTTTC 250

GU808274.1 CCTGACGATTGAAGCGAGGATTTTAAATTATTTGAATTCGAAGGCTTTTC 250

GU808275.1 CCTGACGATTGAAGCGAGGATTTTAAATTATTTGAATTCGAAGGCTTTTC 250

GU808276.1 CCTGACGATTGAAGCGAGGATTTTAAATTATTTGAATTCGAAGGCTTTTC 250

R V K N T N P E L M K K I I P I

GU808256.1 ATAGAGTGAAAAATACAAACCCAGAGTTGATGAAAAAAATTATACCGATA 300

GU808257.1 ATAGAGTGAAAAATACAAACCCAGAGTTGATGAAAAAAATTATACCGATA 300

GU808258.1 ATAGAGTGAAAAATACAAACCCAGAGTTGATGAAAAAAATTATACCGATA 300

GU808259.1 ATAGAGTGAAAAATACAAACCCAGAGTTGATGAAAAAAATTATACCGATA 300

GU808260.1 ATAGAGTGAAAAATACAAACCCAGAGTTGATGAAAAAAATTATACCGATA 300

GU808261.1 ATAGAGTGAAAAATACAAACCCAGAGTTGATGAAAAAAATTATACCGATA 300

GU808262.1 ATAGAGTGAAAAATACAAACCCAGAGTTGATGAAAAAAATTATACCGATA 300

GU808263.1 ATAGAGTGAAAAATACAAACCCAGAGTTGATGAAAAAAATTATACCGATA 300

GU808264.1 ATAGAGTGAAAAATACAAACCCAGAGTTGATGAAAAAAATTATACCGATA 300

GU808265.1 ATAGAGTGAAAAATACAAACCCAGAGTTGATGAAAAAAATTATACCGATA 300

R V K N T H P E L M K K I I P I

GU808266.1 ATAGAGTGAAAAATACACACCCAGAGTTGATGAAAAAAATTATACCGATA 300

GU808267.1 ATAGAGTGAAAAATACAAACCCAGAGTTGATGAAAAAAATTATACCGATA 300

GU808268.1 ATAGAGTGAAAAATACAAACCCAGAGTTGATGAAAAAAATTATACCGATA 300

GU808269.1 ATAGAGTGAAAAATACAAACCCAGAGTTGATGAAAAAAATTATACCGATA 300

GU808270.1 ATAGAGTGAAAAATACAAACCCAGAGTTGATGAAAAAAATTATACCGATA 300

GU808271.1 ATAGAGTGAAAAATACAAACCCAGAGTTGATGAAAAAAATTATACCGATA 300

GU808272.1 ATAGAGTGAAAAATACAAACCCAGAGTTGATGAAAAAAATTATACCGATA 300

GU808273.1 ATAGAGTGAAAAATACAAACCCAGAGTTGATGAAAAAAATTATACCGATA 300

GU808274.1 ATAGAGTGAAAAATACAAACCCAGAGTTGATGAAAAAAATTATACCGATA 300

GU808275.1 ATAGAGTGAAAAATACAAACCCAGAGTTGATGAAAAAAATTATACCGATA 300

GU808276.1 ATAGAGTGAAAAATACAAACCCAGAGTTGATGAAAAAAATTATACCGATA 300

C G N L E D K N L G I S D S D M K

GU808256.1 TGTGGGAATTTGGAAGATAAAAATCTTGGTATCAGCGACAGCGACATGAA 350

GU808257.1 TGTGGGAATTTGGAAGATAAAAATCTTGGTATCAGCGACAGCGACATGAA 350

GU808258.1 TGTGGGAATTTGGAAGATAAAAATCTTGGTATCAGCGACAGCGACATGAA 350

GU808259.1 TGTGGGAATTTGGAAGATAAAAATTTTGGTATCAGCGACAGCGACATGAA 350

GU808260.1 TGTGGGAATTTGGAAGATAAAAATCTTGGTATCAGCGACAGCGACATGAA 350

GU808261.1 TGTGGGAATTTGGAAGATAAAAATCTTGGTATCAGCGACAGCGACATGAA 350

GU808262.1 TGTGGGAATTTGGAAGATAAAAATCTTGGTATCAGCGACAGCGACATGAA 350

GU808263.1 TGTGGGAATTTGGAAGATAAAAATCTTGGTATCAGCGACAGCGACATGAA 350

GU808264.1 TGTGGGAATTTGGAAGATAAAAATCTTGGTATCAGCGACAGCGACATGAA 350

GU808265.1 TGTGGGAATTTGGAAGATAAAAATCTTGGTATCAGCGACAGCGACATGAA 350

C G N L E D K N L G I S D S D M K

GU808266.1 TGTGGGAATTTGGAAGATAAAAATCTTGGTATCAGCGACAGCGACATGAA 350

GU808267.1 TGTGGGAATTTGGAAGATAAAAATCTTGGTATCAGCGACAGCGACATGAA 350

GU808268.1 TGTGGGAATTTGGAAGATAAAAATCTTGGTATCAGCGACAGCGACATGAA 350

GU808269.1 TGTGGGAATTTGGAAGATAAAAATCTTGGTATCAGCGACAGCGACATGAA 350

GU808270.1 TGTGGGAATTTGGAAGATAAAAATCTTGGTATCAGCGACAGCGACATGAA 350

GU808271.1 TGTGGGAATTTGGAAGATAAAAATCTTGGTATCAGCGACAGCGACATGAA 350

GU808272.1 TGTGGGAATTTGGAAGATAAAAATCTTGGTATCAGCGACAGCGACATGAA 350

GU808273.1 TGTGGGAATTTGGAAGATAAAAATCTTGGTATCAGCGACAGCGACATGAA 350

GU808274.1 TGTGGGAATTTGGAAGATAAAAATCTTGGTATCAGCGACAGCGACATGAA 350

GU808275.1 TGTGGGAATTTGGAAGATAAAAATCTTGGTATCAGCGACAGCGACATGAA 350

GU808276.1 TGTGGGAATTTGGAAGATAAAAATCTTGGTATCAGCGACAGCGACATGAA 350

T L L E E V S I V F H V A A K L L

GU808256.1 AACGCTTCTAGAGGAGGTATCCATCGTTTTTCATGTAGCTGCAAAATTGT 400

GU808257.1 AACGCTTCTAGAGGAGGTATCCATCGTTTTTCATGTAGCTGCAAAATTGT 400

GU808258.1 AACGCTTCTAGAGGAGGTATCCATCGTTTTTCATGTAGCTGCAAAATTGT 400

GU808259.1 AACGCTTCTAGAGGAGGTATCCATCGTTTTTCATGTAGCTGCAAAATTGT 400

GU808260.1 AACGCTTCTAGAGGAGGTATCCATCGTTTTTCATGTAGCTGCAAAATTGT 400

GU808261.1 AACGCTTCTAGAGGAGGTATCCATCGTTTTTCATGTAGCTGCAAAATTGT 400

GU808262.1 AACGCTTCTAGAGGAGGTATCCATCGTTTTTCATGTAGCTGCAAAATTGT 400

GU808263.1 AACGCTTCTAGAGGAGGTATCCATCGTTTTTCATGTAGCTGCAAAATTGT 400

GU808264.1 AACGCTTCTAGAGGAGGTATCCATCGTTTTTCATGTAGCTGCAAAATTGT 400

GU808265.1 AACGCTTCTAGAGGAGGTATCCATCGTTTTTCATGTAGCTGCAAAATTGT 400

T L L E E V S I V F H L A A K L L

GU808266.1 AACGCTTCTAGAGGAGGTATCCATCGTTTTTCATTTAGCTGCAAAATTGT 400

GU808267.1 AACGCTTCTAGAGGAGGTATCCATCGTTTTTCATTTAGCTGCAAAATTGT 400

GU808268.1 AACGCTTCTAGAGGAGGTATCCATCGTTTTTCATTTAGCTGCAAAATTGT 400

GU808269.1 AACGCTTCTAGAGGAGGTATCCATCGTTTTTCATTTAGCTGCAAAATTGT 400

GU808270.1 AACGCTTCTAGAGGAGGTATCCATCGTTTTTCATTTAGCTGCAAAATTGT 400

GU808271.1 AACGCTTCTAGAGGAGGTATCCATCGTTTTTCATTTAGCTGCAAAATTGT 400

GU808272.1 AACGCTTCTAGAGGAGGTATCCATCGTTTTTCATTTAGCTGCAAAATTGT 400

GU808273.1 AACGCTTCTAGAGGAGGTATCCATCGTTTTTCATTTAGCTGCAAAATTGT 400

GU808274.1 AACGCTTCTAGAGGAGGTATCCATTGTTTTTCATTTAGCTGCAAAATTGT 400

GU808275.1 AACGCTTCTAGAGGAGGTATCCATCGTTTTTCATTTAGCTGCAAAATTGT 400

GU808276.1 AACGCTTCTAGAGGAGGTATCCATCGTTTTTCATTTAGCTGCAAAATTGT 400

>>>>>>>>>>>>>>>>>>>>>*Nla*III

F K M S L T A A V N I N T K P T

GU808256.1 TATTTAAAATGAGCTTGACTGCAGCAGTCAATATAAATACCAAACCCACT 450

GU808257.1 TATTTAAAATGAGCTTGACTGCAGCAGTCAATATAAATACCAAACCCACT 450

GU808258.1 TATTTAAAATGAGCTTGACTGCAGCAGTCAATATAAATACCAAACCCACT 450

GU808259.1 TATTTAAAATGAGCTTGACTGCAGCAGTCAATATAAATACCAAACCCACT 450

GU808260.1 TATTTAAAATGAGCTTGACTGCAACAGTCAATATAAATACCAAACCCACT 450

GU808261.1 TATTTAAAATGAGCTTGACTGCAGCAGTCAATATAAATACCAAACCCACT 450

GU808262.1 TATTTAAAATGAGCTTGACTGCAGCAGTCAATATAAATACCAAACCCACT 450

GU808263.1 TATTTAAAATGAGCTTGACTGCAGCAGTCAATATAAATACCAAACCCACT 450

GU808264.1 TATTTAAAATGAGCTTGACTGCAGCAGTCAATATAAATACCAAACCCACT 450

GU808265.1 TATTTAAAATGAGCTTGACTGCAGCAGTCAATATAAATACCAAACCCACT 450

F K M S L A A A V N I N T K S T

GU808266.1 TATTTAAAATGAGCTTGGCTGCAGCAGTCAATATAAATACCAAATCCACT 450

GU808267.1 TATTTAAAATGAGCTTGGCTGCAGCAGTCAATATAAATACCAAATCCACT 450

GU808268.1 TATTTAAAATGAGCTTGGCTGCAGCAGTCAATATAAATACCAAATCCACT 450

GU808269.1 TATTTAAAATGAGCTTGGCTGCAGCAGTCAATATAAATACCAAATCCACT 450

GU808270.1 TATTTAAAATGAGCTTGGCTGCAGCAGTCAATATAAATACCAAATCCACT 450

GU808271.1 TATTTAAAATGAGCTTGGCTGCAGCAGTCAATATAAATACCAAATCCACT 450

GU808272.1 TATTTAAAATGAGCTTGGCTGCAGCAGTCAATATAAATACCAAATCCACT 450

GU808273.1 TATTTAAAATGAGCTTGGCTGCAGCAGTCAATATAAATACCAAATCCACT 450

GU808274.1 TATTTAAAATGAGCTTGGCTGCAGCAGTCAATATAAATACCAAATCCACT 450

GU808275.1 TATTTAAAATGAGCTTGGCTGCAGCAGTCAATATAAATACCAAATCCACT 450

GU808276.1 TATTTAAAATGAGCTTGGCTGCAGCAGTCAATATAAATACCAAATCCACT 450

E Q L I A I C K K M R R N P I F I

GU808256.1 GAACAGCTCATAGCGATTTGCAAAAAAATGCGGCGTAATCCCATTTTCAT 500

GU808257.1 GAACAGCTCATAGCGATTTGCAAAAAAATGCGGCGTAATCCCATTTTCAT 500

GU808258.1 GAACAGCTCATAGCGATTTGCAAAAAAATGCGGCGTAATCCCATTTTCAT 500

GU808259.1 GAACAGCTCATAGCGATTTGCAAAAAAATGCGGCGTAATCCCATTTTCAT 500

GU808260.1 GAACAGCTCATAGCGATTTGCAAAAAAATGCGGCGTAATCCCATTTTCAT 500

GU808261.1 GAACAGCTCATAGCGATTTGCAAAAAAATGCAGCGTAATCCCATTTTCAT 500

GU808262.1 GAACAGCTCATAGCGATTTGCAAAAAAATGCGGCGTAATCCCATTTTCAT 500

GU808263.1 GAACAGCTCATAGCGATTTGCAAAAAAATGCGGCGTAATCCCATTTTCAT 500

GU808264.1 GAACAGCTCATAGCGATTTGCAAAAAAATGCGGCGTAATCCCATTTTCAT 500

GU808265.1 GAACAGCTCATAGCGATTTGCAAAAAAATGCGGCGTAATCCCATTTTCAT 500

E Q L I A I C K K M R R N P I F I

GU808266.1 GAACAGCTCATAGCGATTTGCAAAAAAATGCGGCGTAATCCCATTTTCAT 500

GU808267.1 GAACAGCTCATAGCGATTTGCAAAAAAATGCGGCGTAATCCCATTTTCAT 500

GU808268.1 GAACAGCTCATAGCGATTTGCAAAAAAATGCGGCGTAATCCCATTTTCAT 500

GU808269.1 GAACAGCTCATAGCGATTTGCAAAAAAATGCGGCGTAATCCCATTTTCAT 500

GU808270.1 GAACAGCTCATAGCGATTTGCAAAAAAATGCGGCGTAATCCCATTTTCAT 500

GU808271.1 GAACAGCTCATAGCGATTTGCAAAAAAATGCGGCGTAATCCCATTTTCAT 500

GU808272.1 GAACAGCTCATAGCGATTTGCAAAAAAATGCGGCGTGATCCCATTTTCAT 500

GU808273.1 GAACAGCTCATAGCGATTTGCAAAAAAATGCGGCGTAATCCCATTTTCAT 500

GU808274.1 GAACAGCTCATAGCGATTTGCAAAAAAATGCGGCGTAATCCCATTTTCAT 500

GU808275.1 GAACAGCTCATAGCGATTTGCAAAAAAATGCGGCGTAATCCCATTTTCAT 500

GU808276.1 GAACAGCTCATAGCGATTTGCAAAAAAATGCGGCGTAATCCCATTTTCAT 500

<<<<<<<<<<<<<<<<<<

Y V S S A Y S N V N E Q I I D E K

GU808256.1 CTATGTCTCTAGCGCATACAGTAATGTAAATGAACAAATAATCGATGAAA 550

GU808257.1 CTATGTCTCTAGCGCATACAGTAATGTAAATGAACAAATAATCGATGAAA 550

GU808258.1 CTATGTCTCTAGCGCATACAGTAATGTAAATGAACAAATAATCGATGAAA 550

GU808259.1 CTATGTCTCTAGCGCATACAGTAATGTAAATGAACAAATAATCGATGAAA 550

GU808260.1 CTATGTCTCTAGCGCATACAGTAATGTAAATGAACAAATAATCGATGAAA 550

GU808261.1 CTATGTCTCTAGCGCATACAGTAATGTAAATGAACAAATAATCGATGAAA 550

GU808262.1 CTATGTCTCTAGCGCATACAGTAATGTAAATGAACAAATAATCGATGAAA 550

GU808263.1 CTATGTCTCTAGCGCATACAGTAATGTAAATGAACAAATAATCGATGAAA 550

GU808264.1 CTATGTCTCTAGCGCATACAGTAATGTAAATGAACAAATAATCGATGAAA 550

GU808265.1 CTATGTCTCTAGCGCATACAGTAATGTAAATGAACAAATAATCGATGAAA 550

Y V S S A Y S N V N K Q I I D E K

GU808266.1 CTATGTCTCTAGCGCATACAGTAATGTAAATAAACAAATAATCGATGAAA 550

GU808267.1 CTATGTCTCTAGCGCATACAGTAATGTAAATAAACAAATAATCGATGAAA 550

GU808268.1 CTATGTCTCTAGCGCATACAGTAATGTAAATAAACAAATAATCGATGAAA 550

GU808269.1 CTATGTCTCTAGCGCATACAGTAATGTAAATAAACAAATAATCGATGAAA 550

GU808270.1 CTATGTCTCTAGCGCATACAGTAATGTAAATAAACAAATAATCGATGAAA 550

GU808271.1 CTATGTCTCTAGCGCATACAGTAATGTAAATAAACAAATAATCGATGAAA 550

GU808272.1 CTATGTCTCTAGCGCATACAGTAATGTAAATAAACAAATAATCGATGAAA 550

GU808273.1 CTATGTCTCTAGCGCATACAGTAATGTAAATAAACAAATAATCGATGAAA 550

GU808274.1 CTATGTCTCTAGCGCATACAGTAATGTAAATAAACAAATAATCGATGAAA 550

GU808275.1 CTATGTCTCTAGCGCATACAGTAATGTAAATAAACAAATAATCGATGAAA 550

GU808276.1 CTATGTCTCTAGCGCATACAGTAATGTAAATAAACAAATAATCGATGAAA 550

*Fok*I

V Y N T G V P L E T I Y D T L D

GU808256.1 AAGTGTACAACACTGGAGTACCTTTGGAAACTATTTATGATACGCTGGAT 600

GU808257.1 AAGTGTACAACACTGGAGTACCTTTGGAAACTATTTATGATACGCTGGAT 600

GU808258.1 AAGTGTACAACACTGGAGTACCTTTGGAAACTATTTATGATACGCTGGAT 600

GU808259.1 AAGTGTACAACACTGGAGTACCTTTGGAAACTATTTATGATACGCTGGAT 600

GU808260.1 AAGTGTACAACACTGGAGTACCTTTGGAAACTATTTATGATACGCTGGAT 600

GU808261.1 AAGTGTACAACACTGGAGTACCTTTGGAAACTATTTATGATACGCTGGAT 600

GU808262.1 AAGTGTACAACACTGGAGTACCTTTGGAAACTATTTATGATACGCTGGAT 600

GU808263.1 AAGTGTACAACACTGGAGTACCTTTGGAAACTATTTATGATACGCTGGAT 600

GU808264.1 AAGTGTACAACACTGGAGTACCTTTGGAAACTATTTATGATACGCTGGAT 600

GU808265.1 AAGTGTACAACACTGGAGTACCTTTGGAAACTATTTATGATACGCTGGAT 600

V Y S T G V P L E T I Y D T L D

GU808266.1 AAGTGTACAGCACTGGAGTACCTTTGGAAACTATTTATGATACGCTGGAT 600

GU808267.1 AAGTGTACAGCACTGGAGTACCTTTGGAAACTATTTATGATACGCTGGAT 600

GU808268.1 AAGTGTACAGCACTGGAGTACCTTTGGAAACTATTTATGATACGCTGGAT 600

GU808269.1 AAGTGTACAGCACTGGAGTACCTTTGGAAACTATTTATGATACGCTGGAT 600

GU808270.1 AAGTGTACAGCACTGGAGTACCTTTGGAAACTATTTATGATACGCTGGAT 600

GU808271.1 AAGTGTACAGCACTGGAGTACCTTTGGAAACTATTTATGATACGCTGGAT 600

GU808272.1 AAGTGTACAGCACTGGAGTACCTTTGGAAACTATTTATGATACGCTGGAT 600

GU808273.1 AAGTGTACAGCACTGGAGTACCTTTGGAAACTATTTATGATACGCTGGAT 600

GU808274.1 AAGTGTACAGCACTGGAGTACCTTTGGAAACTATTTATGATACGCTGGAT 600

GU808275.1 AAGTGTACAGCACTGGAGTACCTTTGGAAACTATTTATGATACGCTGGAT 600

GU808276.1 AAGTGTACAGCACTGGAGTACCTTTGGAAACTATTTATGATACGCTGGAT 600

T E N T R I T D I F L D K R P N T

GU808256.1 ACAGAAAATACACGAATAACGGATATTTTTTTAGATAAAAGACCAAATAC 650

GU808257.1 ACAGAAAATACACGAATAACGGATATTTTTTTAGATAAAAGACCAAATAC 650

GU808258.1 ACAGAAAATACACGAATAACGGATATTTTTTTAGATAAAAGACCAAATAC 650

GU808259.1 ACAGAAAATACACGAATAACGGATATTTTTTTAGATAAAAGACCAAATAC 650

GU808260.1 ACAGAAAATACACGAATAACGGATATTTTTTTAGATAAAAGACCAAATAC 650

GU808261.1 ACAGAAAATACACGAATAACGGATATTTTTTTAGATAAAAGACCAAATAC 650

GU808262.1 ACAGAAAATACACGAATAACGGATATTTTTTTAGATAAAAGACCAAATAC 650

GU808263.1 ACAGAAAATACACGAATAACGGATATTTTTTTAGATAAAAGACCAAATAC 650

GU808264.1 ACAGAAAATACACGAATAACGGATATTTTTTTAGATAAAAGACCAAATAC 650

GU808265.1 ACAGAAAATACACGAATAACGGATATTTTTTTAGATAAAAGACCAAATAC 650

A K N T R L M D I F L D K R P N T

GU808266.1 GCAAAAAATACACGACTTATGGATATTTTTTTAGATAAAAGACCAAATAC 650

GU808267.1 GCAAAAAATACACGACTTATGGATATTTTTTTAGATAAAAGACCAAATAC 650

GU808268.1 GCAAAAAATACACGACTTATGGATATTTTTTTAGATAAAAGACCAAATAC 650

GU808269.1 GCAAAAAATACACGACTTATGGATATTTTTTTAGATAAAAGACCAAATAC 650

GU808270.1 GCAAAAAATACACGACTTATGGATATTTTTTTAGATAAAAGACCAAATAC 650

GU808271.1 GCAAAAAATACACGACTTATGGATATTTTTTTAGATAAAAGACCAAATAC 650

GU808272.1 GCAAAAAATACACGACTTATGGATATTTTTTTAGATAAAAGACCAAATAC 650

GU808273.1 GCAAAAAATACACGACTTATGGATATTTTTTTAGATAAAAGACCAAATAC 650

GU808274.1 GCAAAAAATACACGACTTATGGATATTTTTTTAGATAAAAGACCAAATAC 650

GU808275.1 GCAAAAAATACACGACTTATGGATATTTTTTTAGATAAAAGACCAAATAC 650

GU808276.1 GCAAAAAATACACGACTTATGGATATTTTTTTAGATAAAAGACCAAATAC 650

Y T Y S K A L A E V V V E K E F D

GU808256.1 GTATACCTATTCAAAAGCTCTTGCAGAAGTAGTAGTTGAAAAAGAATTTG 700

GU808257.1 GTATACCTATTCAAAAGCTCTTGCAGAAGTAGTAGTTGAAAAAGAATTTG 700

GU808258.1 GTATACCTATTCAAAAGCTCTTGCAGAAGTAGTAGTTGAAAAAGAATTTG 700

GU808259.1 GTATACCTATTCAAAAGCTCTTGCAGAAGTAGTAGTTGAAAAAGAATTTG 700

GU808260.1 GTATACCTATTCAAAAGCTCTTGCAGAAGTAGTAGTTGAAAAAGAATTTG 700

GU808261.1 GTATACCTATTCAAAAGCTCTTGCAGAAGTAGTAGTTGAAAAAGAATTTG 700

GU808262.1 GTATACCTATTCAAAAGCTCTTGCAGAAGTAGTAGTTGAAAAAGAATTTG 700

GU808263.1 GTATACCTATTCAAAAGCTCTTGCAGAAGTAGTAGTTGAAAAAGAATTTG 700

GU808264.1 GTATACCTATTCAAAAGCTCTTGCAGAAGTAGTAGTTGAAAAAGAATTTG 700

GU808265.1 GTATACCTATTCAAAAGCTCTTGCAGAAGTAGTAGTTGAAAAAGAATTTG 700

Y T Y S K A L A E V L V E N E F D

GU808266.1 GTATACCTATTCAAAAGCTCTTGCAGAAGTACTAGTTGAAAATGAATTTG 700

GU808267.1 GTATACCTATTCAAAAGCTCTTGCAGAAGTACTAGTTGAAAAAGAATTTA 700

GU808268.1 GTATACCTATTCAAAAGCTCTTGCAGAAGTACTAGTTGAAAAAGAATTTG 700

GU808269.1 GTATACCTATTCAAAAGCTCTTGCAGAAGTACTAGTTGAAAAAGAATTTG 700

GU808270.1 GTATACCTATTCAAAAGCTCTTGCAGAAGTACTAGTTGAAAATGAATTTG 700

GU808271.1 GTATACCTATTCAAAAGCTCTTGCAGAAGTACTAGTTGAAAAAGAATTTG 700

GU808272.1 GTATACCTATTCAAAAGCTCTTGCAGAAGTACTAGTTGAAAAAGAATTTG 700

GU808273.1 GTATACCTATTCAAAAGCTCTTGCAGAAGTACTAGTTGAAAAAGAATTTG 700

GU808274.1 GTATACCTATTCAAAAGCTCTTGCAGAAGTACTAGTTGAAAAAGAATTTG 700

GU808275.1 GTATACCTATTCAAAAGCTCTTGCAGAAGTACTAGTTGAAAATGAATTTG 700

GU808276.1 GTATACCTATTCAAAAGCTCTTGCAGAAGTACTAGTTGAAAATGAATTTG 700

E S A A I V R P S I I V S S I R

GU808256.1 ATGAATCAGCAGCCATTGTTCGGCCTTCGATAATTGTGTCTTCGATTCGG 750

GU808257.1 ATGAATCAGCAGCCATTGTTCGGCCTTCGATAATTGTGTCTTCGATTCGG 750

GU808258.1 ATGAATCAGCAGCCATTGTTCGGCCTTCGATAATTGTGTCTTCGATTCGG 750

GU808259.1 ATGAATCAGCAGCCATTGTTCGGCCTTCGATAATTGTGTCTTCGATTCGG 750

GU808260.1 ATGAATCAGCAGCCATTGTTCGGCCTTCGATAATTGTGTCTTCGATTCGG 750

GU808261.1 ATGAATCAGCAGCCATTGTTCGGCCTTCGATAATTGTGTCTTCGATTCGG 750

GU808262.1 ATGAATCAGCAGCCATTGTTCGGCCTTCGATAATTGTGTCTTCGATTCGG 750

GU808263.1 ATGAATCAGCAGCCATTGTTCGGCCTTCGATAATTGTGTCTTCGATTCGG 750

GU808264.1 ATGAATCAGCAGCCATTGTTCGGCCTTCGATAATTGTGTCTTCGATTCGG 750

GU808265.1 ATGAATCAGCAGCCATTGTTCGGCCTTCGATAATTGTGTCTTCGATTCGG 750

E S A A I V R P S I I A S S I R

GU808266.1 ACGAATCAGCAGCCATTGTTCGGCCTTCGATAATTGCGTCTTCGATTCGG 750

GU808267.1 ACGAATCAGCAGCCATTGTTCGGCCTTCGATAATTGCGTCTTCGATTCGG 750

GU808268.1 ACGAATCAGCAGCCATTGTTCGGCCTTCGATAATTGCGTCTTCGATTCGG 750

GU808269.1 ACGAATCAGCAGCCATTGTTCGGCCTTCGATAATTGCGTCTTCGATTCGG 750

GU808270.1 ACGAATCAGCAGCCATTGTTCGGCCTTCGATAATTGCGTCTTCGATTCGG 750

GU808271.1 ACGAATCAGCAGCCATTGTTCGGCCTTCGATAATTGCGTCTTCGATTCGG 750

GU808272.1 ACGAATCAGCAGCCATTGTTCGGCCTTCGATAATTGCGTCTTCGATTCGG 750

GU808273.1 ACGAATCAGCAGCCATTGTTCGGCCTTCGATAATTGCGTCTTCGATTCGG 750

GU808274.1 ACGAATCAGCAGCCATTGTTCGGCCTTCGATAATTGCGTCTTCGATTCGG 750

GU808275.1 ACGAATCAGCAGCCATTGTTCGGCCTTCGATAATTGCGTCTTCGATTCGG 750

GU808276.1 ACGAATCAGCAGCCATTGTTCGGCCTTCGATAATTGCGTCTTCGATTCGG 750

>>>>>>>>>>

E P I P G W L S G S H G F P R V V

GU808256.1 GAACCCATACCGGGATGGTTGAGCGGTTCGCACGGATTCCCTAGGGTAGT 800

GU808257.1 GAACCCATACCGGGATGGTTGAGCGGTTCGCACGGATTCCCTAGGGTAGT 800

GU808258.1 GAACCCATACCGGGATGGTTGAGCGGTTCGCACGGATTCCCTAGGGTAGT 800

GU808259.1 GAACCCATACCGGGATGGTTGAGCGGTTCGCACGGATTCCCTAGGGTAGT 800

GU808260.1 GAACCCATACCGGGATGGTTGAGCGGTTCGCACGGATTCCCTAGGGTAGT 800

GU808261.1 GAACCCATACCGGGATGGTTGAGCGGTTTGCACGGATTCCCTAGGGTAGT 800

GU808262.1 GAACCCATACCGGGATGGTTGAGCGGTTCGCACGGATTCCCTAGGGTAGT 800

GU808263.1 GAACCCATACCGGGATGGTTGAGCGGTTCGCACGGATTCCCTAGGGTAGT 800

GU808264.1 GAACCCATACCGGGATGGTTGAGCGGTTCGCACGGATTCCCTAGGGTAGT 800

GU808265.1 GAACCCATACCGGGATGGTTGAGCGGTTCGCACGGATTCCCTAGGGTAGT 800

E P I P G W L S G S H G F P R V V

GU808266.1 GAACCCATACCGGGATGGTTGAGCGGTTCGCATGGATTCCCTAGGGTAGT 800

GU808267.1 GAACCCATACCGGGATGGTTGAGCGGTTCGCACGGATTCCCTAGGGTAGT 800

GU808268.1 GAACCCATACCGGGATGGTTGAGCGGTTCGCACGGATTCCCTAGGGTAGT 800

GU808269.1 GAACCCATACCGGGATGGTTGAGCGGTTCGCACGGATTCCCTAGGGTAGT 800

GU808270.1 GAACCCATACCGGGATGGTTGAGCGGTTCGCACGGATTCCCTAGGGTAGT 800

GU808271.1 GAACCCATACCGGGATGGTTGAGCGGTTCGCACGGATTCCCTAGGGTAGT 800

GU808272.1 GAACCCATACCGGGATGGTTGAGCGGTTCGCACGGATTCCCTAGGGTAGT 800

GU808273.1 GAACCCATACCGGGATGGTTGAGCGGTTCGCACGGATTCCCTAGGGTAGT 800

GU808274.1 GAACCCATACCGGGATGGTTGAGCGGTTCGCACGGATTCCCTAGGGTAGT 800

GU808275.1 GAACCCATACCGGGATGGTTGAGCGGTTCGCACGGATTCCCTAGGGTAGT 800

GU808276.1 GAACCCATACCGGGATGGTTGAGCGGTTCGCACGGATTCCCTAGGGTAGT 800

>>>>>>>>>

G A A C K G L L L R W H G D G T V

GU808256.1 AGGAGCAGCATGCAAGGGGCTCCTCTTGCGGTGGCATGGGGACGGTACAG 850

GU808257.1 AGGAGCAGCATGCAAGGGGCTCCTCTTGCGGTGGCATGGGGACGGTACAG 850

GU808258.1 AGGAGCAGCATGCAAGGGGCTCCTCTTGCGGTGGCATGGGGACGGTACAG 850

GU808259.1 AGGAGCAGCATGCAAGGGGCTCCTCTTGCGGTGGCATGGGGACGGTACAG 850

GU808260.1 AGGAGCAGCATGCAAGGGGCTCCTCTTGCGGTGGCATGGGGACGGTACAG 850

GU808261.1 AGGAGCAGCATGCAAGGGGCTCCTCTTGCGGTGGCATGGGGACGGTACAG 850

GU808262.1 AGGAGCAGCATGCAAGGGGCTCCTCTTGCGGTGGCATGGGGACGGTACAG 850

GU808263.1 AGGAGCAGCATGCAAGGGGCTCCTCTTGCGGTGGCATGGGGACGGTACAG 850

GU808264.1 AGGAGCAGCATGCAAGGGGCTCCTCTTGCGGTGGCATGGGGACGGTACAG 850

GU808265.1 AGGAGCAGCATGCAAGGGGCTCCTCTTGCGGTGGCATGGGGACGGTACAG 850

E A A C K G L L L R W H G D G T V

GU808266.1 AGAAGCAGCATGCAAGGGGCTCCTCTTGCGGTGGCATGGGGACGGTACAG 850

GU808267.1 AGAAGCAGCATGCAAGGGGCTCCTCTTGCGGTGGCATGGGGACGGTACAG 850

GU808268.1 AGAAGCAGCATGCAAGGGGCTCCTCTTGCGGTGGCATGGGGACGGTACAG 850

GU808269.1 AGAAGCAGCATGCAAGGGGCTCCTCTTGCGGTGGCATGGGGACGGTACAG 850

GU808270.1 AGAAGCAGCATGCAAGGGGCTCCTCTTGCGGTGGCATGGGGACGGTACAG 850

GU808271.1 AGAAGCAGCATGCAAGGGGCTCCTCTTGCGGTGGCATGGGGACGGTACAG 850

GU808272.1 AGAAGCAGCATGCAAGGGGCTCCTCTTGCGGTGGCATGGGGACGGTACAG 850

GU808273.1 AGAAGCAGCATGCAAGGGGCTCCTCTTGCGGTGGCATGGGGACGGTACAG 850

GU808274.1 AGAAGCAGCACGCAAGGGGCTCCTCTTGCGGTGGCATGGGGACGGTACAG 850

GU808275.1 AGAAGCAGCATGCAAGGGGCTCCTCTTGCGGTGGCATGGGGACGGTACAG 850

GU808276.1 AGAAGCAGCATGCAAGGGGCTCCTCTTGCGGTGGCATGGGGACGGTACAG 850

V C D L I P V D H V A N L I I A

GU808256.1 TTGTCTGCGACCTTATACCTGTAGACCACGTTGCAAACCTCATCATTGCA 900

GU808257.1 TTGTCTGCGACCTTATACCTGTAGACCACGTTGCAAACCTCATCATTGCA 900

GU808258.1 TTGTCTGCGACCTTATACCTGTAGACCACGTTGCAAACCTCATCATTGCA 900

GU808259.1 TTGTCTGCGACCTTATACCTGTAGACCACGTTGCAAACCTCATCATTGCA 900

GU808260.1 TTGTCTGCGACCTTATACCTGTAGACCACGTTGCAAACCTCATCATTGCA 900

GU808261.1 TTGTCTGCGACCTTATACCTGTAGACCACGTTGCAAACCTCATCATTGCA 900

GU808262.1 TTGTCTGCGACCTTATACCTGTAGACCACGTTGCGAACCTCATCATTGCA 900

GU808263.1 TTGTCTGCGACCTTATACCTGTAGACCACGTTGCGAACCTCATCATTGCA 900

GU808264.1 TTGTCTGCGACCTTATACCTGTAGACCACGTTGCGAACCTCATCATTGCA 900

GU808265.1 TTGTCTGCGACCTTATACCTGTAGACCACGTTGCGAACCTCATCATTGCA 900

A F D I I P V D H V A N L I I A

GU808266.1 TTGCCTTCGACATTATACCTGTAGACCACGTTGCGAACCTCATAATTGCA 900

GU808267.1 TTGCCTTCGACATTATACCTGTAGACCACGTTGCGAACCTCATAATTGCA 900

GU808268.1 TTGCCTTCGACATTATACCTGTAGACCACGTTGCGAACCTCATAATTGCA 900

GU808269.1 TTGCCTTCGACATTATACCTGTAGACCACGTTGCGAACCTCATAATTGCA 900

GU808270.1 TTGCCTTCGACATTATACCTGTAGACCACGTTGCGAACCTCATAATTGCA 900

GU808271.1 TTGCCTTCGACATTATACCTGTAGACCACGTTGCGAACCTCATAATTGCA 900

GU808272.1 TTGCCTTCGACATTATACCTGTAGACCACGTTGCGAACCTCATAATTGCA 900

GU808273.1 TTGCCTTCGACATTATACCTGTAGACCACGTTGCGAACCTCATAATTGCA 900

GU808274.1 TTGCCTTCGACATTATACCTGTAGACCACGTTGCGAACCTCATAATTGCA 900

GU808275.1 TTGCCTTCGACATTATACCTGTAGACCACGTTGCGAACCTCATAATTGCA 900

GU808276.1 TTGCCTTCGACATTATACCTGTAGACCACGTTGCGAACCTCATAATTGCA 900

*Taq*I <<<<<<<<<<<<<<<<<<<<

A A W E S N E R R L M G N K G V K

GU808256.1 GCAGCATGGGAATCCAATGAAAGACGGTTAATGGGCAACAAAGGAGTCAA 950

GU808257.1 GCAGCATGGGAATCCAATGAAAGACGGTTAATGGGCAACAAAGGAGTCAA 950

GU808258.1 GCAGCATGGGAATCCAATGAAAGACGGTTAATGGGCAACAAAGGAGTCAA 950

GU808259.1 GCAGCATGGGAATCCAATGAAAGACGGTTAATGGGCAACAAAGGAGTCAA 950

GU808260.1 GCAGCATGGGAATCCAATGAAAGACGGTTAATGGGCAACAAAGGAGTCAA 950

GU808261.1 GCAGCATGGGAATCCAATGAAAGACGGTTAATGGGCAACAAAGGAGTCAA 950

GU808262.1 GCAGCATGGGAATCCAATGAAAGACGGTTAATGGGCAACAAAGGAGTCAA 950

GU808263.1 GCAGCATGGGAATCCAATGAAAGACGGTTAATGGGCAACAAAGGAGTCAA 950

GU808264.1 GCAGCATGGGAATCCAATGAAAGACGGTTAATGGGCAACAAAGGAGTCAA 950

GU808265.1 GCAGCATGGGAATCCAATGAAAGGCGGTTAATGGGCAACAAAGGAGTCAA 950

A A W E S N E R R L M G N K G V K

GU808266.1 GCAGCATGGGAATCCAACGAAAGACGGTTAATGGGCAACAAAGGAGTCAA 950

GU808267.1 GCAGCATGGGAATCCAACGAAAGACGGTTAATGGGCAACAAAGGAGTCAA 950

GU808268.1 GCAGCATGGGAATCCAACGAAAGACGGTTAATGGGCAACAAAGGAGTCAA 950

GU808269.1 GCAGCATGGGAATCCAACGAAAGACGGTTAATGGGCAACAAAGGAGTCAA 950

GU808270.1 GCAGCATGGGAATCCAACGAAAGACGGTTAATGGGCAACAAAGGAGTCAA 950

GU808271.1 GCAGCATGGGAATCCAACGAAAGACGGTTAATGGGCAACAAAGGAGTCAA 950

GU808272.1 GCAGCATGGGAATCCAACGAAAGACGGTTAATGGGCAACAAAGGAGTCAA 950

GU808273.1 GCAGCATGGGAATCCAACGAAAGACGGTTAATGGGCAACAAAGGAGTCAA 950

GU808274.1 GCAGCATGGGAATCCAACGAAAGACGGTTAATGGGCAACAAAGGAGTCAA 950

GU808275.1 GCAGCATGGGAATCCAACGAAAGACGGTTAATTGGCAACAAAGGAGTCAA 950

GU808276.1 GCAGCATGGGAATCCAACGAAAGACGGTTAATGGGCAACAAAGGAGTCAA 950

>>>>>>>>>>>>>>>>>>

V Y N C C S S L R N P I D V I T V

GU808256.1 GGTATATAACTGTTGTTCAAGCCTACGGAACCCAATAGACGTGATCACCG 1000

GU808257.1 GGTATATAACTGTTGTTCAAGCCTACGGAACCCAATAGACGTGATCACCG 1000

GU808258.1 GGTATATAACTGTTGTTCAAGCCTACGGAACCCAATAGACGTGATCACCG 1000

GU808259.1 GGTATATAACTGTTGTTCAAGCCTACGGAACCCAATAGACGTGATCACCG 1000

GU808260.1 GGTATATAACTGTTGTTCAAGCCTACGGAACCCAATAGACGTGATCACCG 1000

GU808261.1 GGTATATAACTGTTGTTCAAGCCTACGGAACCCAATAGACGTGATCACCG 1000

GU808262.1 GGTATATAACTGTTGTTCAAGCCTACGGAACCCAATAGACGTGATCACCG 1000

GU808263.1 GGTATATAACTGTTGTTCAAGCCTACGGAAACCAATAGACGTGATCACCG 1000

GU808264.1 GGTATATAACTGTTGTTCAAGCCTACGGAACCCAATAGACGTGATCACCG 1000

GU808265.1 GGTATATAACTGTTGTTCAAGCCTACGGAACCCAATAGACGTGATCACCG 1000

V Y N C C S G L R N P I D V S T V

GU808266.1 GGTTTACAACTGTTGTTCGGGCCTACGGAACCCAATAGACGTGAGCACCG 1000

GU808267.1 GGTTTACAACTGTTGTTCGGGCCTACGGAACCCAATAGACGTGAGCACCG 1000

GU808268.1 GGTTTACAACTGTTGTTCGGGCCTACGGAACCCAATAGACGTGAGCACCG 1000

GU808269.1 GGTTTACAACTGTTGTTCGGGCCTACGGAACCCAATAGACGTGAGCACCG 1000

GU808270.1 GGTTTACAACTGTTGTTCGGGCCTACGGAACCCAATAGACGTGAGCACCG 1000

GU808271.1 GGTTTACAACTGTTGTTCGGGCCTACGGAACCCAATAGACGTGAGCACCG 1000

GU808272.1 GGTTTACAACTGTTGTTCGGGCCTACGGAACCCAATAGACGTGAGCACCG 1000

GU808273.1 GGTTTACAACTGTTGTTCGGGCCTACGGAACCCAATAGACGTGAGCACCG 1000

GU808274.1 GGTTTACAACTGTTGTTCGGGCCTACGGAACCCAATAGACGTGAGCACCG 1000

GU808275.1 GGTTTACAACTGTTGTTCGGGCCTACGGAACCCAATAGACGTGAGCACCG 1000

GU808276.1 GGTTTACAACTGTTGTTCGGGCCTACGGAACCCAATAGACGTGAGCACCG 1000

>>> NdeII

V K T C I K Y R K Y F G T R T M

GU808256.1 TAGTTAAAACTTGCATAAAATACAGGAAATATTTTGGAACTCGCACCATG 1050

GU808257.1 TAGTTAAAACTTGCATAAAATACAGGAAATATTTTGGAACTCGCACCATG 1050

GU808258.1 TAGTTAAAACTTGCATAAAATACAGGAAATATTTTGGAACTCGCACCATG 1050

GU808259.1 TAGTTAAAACTTGCATAAAATACAGGAAATATTTTGGAACTCGCACCATG 1050

GU808260.1 TAGTTAAAACTTGCATAAAATACAGGAAATATTTTGGAACTCGCACCATG 1050

GU808261.1 TAGTTAAAACTTGCATAAAATACAGGAAATATTTTGGAACTCGCACCATG 1050

GU808262.1 TAGTTAAAACTTGCATAAAATACAGGAAATATTTTGGAACTCGCACCATG 1050

GU808263.1 TAGTTAAAACTTGCATAAAATACAGGAAATATTTTGGAACTCGCACCATG 1050

GU808264.1 TAGTTAAAACTTGCATAAAATACAGGAAATATTTTGGAACTCGCACCATG 1050

GU808265.1 TAGTTAAAACTTGCATAAAATACAGGAAATATTTTGGAACTCGCACCATG 1050

M N T C L K Y R K Y F G T R T M

GU808266.1 TAATGAACACTTGCTTAAAATACAGGAAATATTTTGGAACTCGCACCATG 1050

GU808267.1 TAATGAACACTTGCTTAAAATACAGGAAATATTTTGGAACTCGCACCATG 1050

GU808268.1 TAATGAACACTTGCTTAAAATACAGGAAATATTTTGGAACTCGCACCATG 1050

GU808269.1 TAATGAACACTTGCTTAAAATACAGGAAATATTTTGGAACTCGCACCATG 1050

GU808270.1 TAATGAACACTTGCTTAAAATACAGGAAATATTTTGGAACTCGCACCATG 1050

GU808271.1 TAATGAACACTTGCTTAAAATACAGGAAATATTTTGGAACTCGCACCATG 1050

GU808272.1 TAATGAACACTTGCTTAAAATACAGGAAATATTTTGGAACTCGCACCATG 1050

GU808273.1 TAATGAACACTTGCTTAAAATACAGGAAATATTTTGGAACTCGCACCATG 1050

GU808274.1 TAATGAACACTTGCTTAAAATACAGGAAATATTTTGGAACTCGCACCATG 1050

GU808275.1 TAATGAACACTTGCTTAAAATACAGGAAATATTTTGGAACTCGCACCATG 1050

GU808276.1 TAATGAACACTTGCTTAAAATACAGGAAATATTTTGGAACTCGCACCATG 1050

*Mse*I <<<<<<<<<<<<<<<<<<<<<<<<<<

S I F T P R F I M K K N Y F I Y K

GU808256.1 TCCATATTTACCCCACGATTTATTATGAAAAAGAATTACTTTATCTACAA 1100

GU808257.1 TCCATATTTACCCCACGATTTATTATGAAAAAGAATTACTTTATCTACAA 1100

GU808258.1 TCCATATTTACCCCACGATTTATTATGAAAAAGAATTACTTTATCTACAA 1100

GU808259.1 TCCATATTTACCCCACGATTTATTATGAAAAAGAATTACTTTATCTACAA 1100

GU808260.1 TCCATATTTACCCCACGATTTATTATGAAAAAGAATTACTTTATCTACAA 1100

GU808261.1 TCCATATTTACCCCACGATTTATTATGAAAAAGAATTACTTTATCTACAA 1100

GU808262.1 TCCATATTTACCCCACGATTTATTATGAAAAAGAATTACTTTATCTACAA 1100

GU808263.1 TCCATATTTACCCCACGATTTATTATGAAAAAGAATTACTTTATCTACAA 1100

GU808264.1 TCCATATTTACCCCACGATTTATTATGAAGAAGAATTACTTTATCTACAA 1100

GU808265.1 TCCATATTTACCCCACGATTTATTATGAAGAAGAATTACTTTATCTACAA 1100

S I I T P R F I M K K N Y F L Y K

GU808266.1 TCCATATATACCCCACGATTTATTATGAAAAAGAATTACTTTCTCTACAA 1100

GU808267.1 TCCATAATTACCCCACGATTTATTATGAAAAAGAATTACTTTCTCTACAA 1100

GU808268.1 TCCATAATTACCCCACGATTTATTATGAAAAAGAATTACTTTCTCTACAA 1100

GU808269.1 TCCATAATTACCCCACGATTTATTATGAAAAAGAATTACTTTCTCTACAA 1100

GU808270.1 TCCATAATTACCCCACGATTTATTATGAAAAAGAATTACTTTCTCTACAA 1100

GU808271.1 TCCATAATTACCCCACGATTTATTATGAAAAAGAATTACTTTCTCTACAA 1100

GU808272.1 TCCATAATTACCCCACGATTTATTATGAAAAAGAATTACTTTCTCTACAA 1100

GU808273.1 TCCATAATTACCCCACGATTTATTATGAAAAAGAATTACTTTCTCTATAA 1100

GU808274.1 TCCATAATTACCCCACGATTTATTATGAAAAAGAATTACTTTCTCTATAA 1100

GU808275.1 TCCATAATTACCCCACGATTTATTATGAAAAAGAATTACTTTCTCTACAA 1100

GU808276.1 TCCATAATTACCCCACGATTTATTATGAAAAAGAATTACTTTCTCTACAA 1100

L L Y F T Y H T I P A A I I D G F

GU808256.1 ATTGTTGTACTTCACCTACCACACAATACCGGCAGCTATAATAGACGGCT 1150

GU808257.1 ATTGTTGTACTTCACCTACCACACAATACCGGCAGCTATAATAGACGGCT 1150

GU808258.1 ATTGTTGTACTTCACCTACCACACAATACCGGCAGCTATAATAGACGGCT 1150

GU808259.1 ATTGTTGTACTTCACCTACCACACAATACCGGCAGCTATAATAGACGGCT 1150

GU808260.1 ATTGTTGTACTTCACCTACCACACAATACCGGCAGCTATAATAGACGGCT 1150

GU808261.1 ATTGTTGTACTTCACCTACCACACAATACCGGCAGCTATAATAGACGGCT 1150

GU808262.1 ATTGTTGTACTTCACCTACCACACAATACCGGCAGCTATAATAGACGGCT 1150

GU808263.1 ATTGTTGTACTTCACCTACCACACAATACCGGCAGCTATAATAGACGGCT 1150

GU808264.1 ATTGTTGTACTTCACCTACCACACAATACCGGCAGCTATAATAGACGGCT 1150

GU808265.1 ATTGTTGTACTTCACCTACCACACAATACCGGCAGCTATAATAGACGGCT 1150

L L Y F T Y H T I P A A I I D G F

GU808266.1 ATTGTTGTACTTCACCTACCACACAATACCGGCAGCTATAATAGACGGCT 1150

GU808267.1 ATTGCTGTACTTCACCTACCACACAATACCGGCAGCTATAATAGACGGCT 1150

GU808268.1 ATTGTTGTACTTCACCTACCACACAATACCGGCAGCTATAATAGACGGCT 1150

GU808269.1 ATTGTTGTACTTCACCTACCACACAATACCGGCAGCTATAATAGACGGCT 1150

GU808270.1 ATTGTTGTACTTCACCTACCACACAATACCGGCAGCTATAATAGACGGCT 1150

GU808271.1 ATTGTTGTACTTCACCTACCACACAATACCGGCAGCTATAATAGACGGCT 1150

GU808272.1 ATTGTTGTACTTCACCTACCACACAATACCGGCAGCTATAATAGACGGCT 1150

GU808273.1 ATTGTTGTACTTCACCTACCACACAATACCGGCAGCTATAATAGACGGCT 1150

GU808274.1 ATTGTTGTACTTCACCTACCACACAATACCGGCAGCTATAATAGACGGCT 1150

GU808275.1 ATTGTTGTACTTCACCTACCACACAATACCGGCAGCTATAATAGACGGCT 1150

GU808276.1 ATTGTTGTACTTCACCTACCACACAATACCGGCAGCTATAATAGACGGCT 1150

F W L T G R T P I M L K T L D K

GU808256.1 TCTTCTGGCTCACTGGACGGACTCCAATAATGCTGAAGACCCTGGACAAA 1200

GU808257.1 TCTTCTGGCTCACTGGACGGACTCCAATAATGCTGAAGACCCTGGACAAA 1200

GU808258.1 TCTTCTGGCTCACTGGACGGACTCCAATAATGCTGAAGACCCTGGACAAA 1200

GU808259.1 TCTTCTGGCTCACTGGACGGACTCCAATAATGCTGAAGACCCTGGACAAA 1200

GU808260.1 TCTTCTGGCTCACTGGACGGACTCCAATAATGCTGAAGACCCTGGACAAA 1200

GU808261.1 TCTTCTGGCTCACTGGACGGACTCCAATAATGCTGAAGACCCTGGACAAA 1200

GU808262.1 TCTTCTGGCTCACTGGACGGACTCCAATAATGCTGAAGACCCTGGACAAA 1200

GU808263.1 TCTTCTGGCTCACTGGACGGACTCCAATAATGCTGAAGACCCTGGACAAA 1200

GU808264.1 TCTTCTGGCTCACTGGACGGACTCCAATAATGCTGAAGACCCTGGACAAA 1200

GU808265.1 TCTTCTGGCTCACTGGACGGACTCCAATAATGCTGAAGACCCTGGACAAA 1200

F W L T G R T P M M L N T L H K

GU808266.1 TTTTCTGGCTCACTGGGCGGACTCCAATGATGCTGAACACCCTGCACAAA 1200

GU808267.1 TTTTCTGGCTCACTGGACGGACTCCAATGATGCTGAACACCCTGCACAAA 1200

GU808268.1 TTTTCTGGCTCACTGGACGGACTCCAATGATGCTGAACACCCTGCACAAA 1200

GU808269.1 TTTTCTGGCTCACTGGACGGACTCCAATGATGCTGAACACCCTGCACAAA 1200

GU808270.1 TTTTCTGGCTCACTGGACGGACTCCAATGATGCTGAACACCCTGCACAAA 1200

GU808271.1 TTTTCTGGCTCACTGGACGGACTCCAATGATGCTGAACACCCTGCACAAA 1200

GU808272.1 TTTTCTGGCTCACTGGACGGACTCCAATGATGCTGAACACCCTGCACAAA 1200

GU808273.1 TTTTCTGGCTCACTGGACGGACTCCAATGATGCTGAACACCCTGCACAAA 1200

GU808274.1 TTTTCTGGCTCACTGGACGGACTCCAATGATGCTGAACACCCTGCACAAA 1200

GU808275.1 TTTTCTGGCTCACTGGGCGGACTCCAATGATGCTGAACACCCTGCACAAA 1200

GU808276.1 TTTTCTGGCTCACTGGACGGACTCCAATGATGCTGAACACCCTGCACAAA 1200

L S K I S S V L E Y F T H H Q F I

GU808256.1 CTCAGCAAAATCTCTTCTGTCCTGGAGTACTTCACGCACCACCAATTTAT 1250

GU808257.1 CTCAGCAAAATCTCTTCTGTCCTGGAGTACTTCACGCACCACCAATTTAT 1250

GU808258.1 CTCAGCAAAATCTCTTCTGTCCTGGAGTACTTCACGCACCACCAATTTAT 1250

GU808259.1 CTCAGCAAAATCTCTTCTGTCCTGGAGTACTTCACGCACCACCAATTTAT 1250

GU808260.1 CTCAGCAAAATCTCTTCTGTCCTGGAGTACTTCACGCACCACCAATTTAT 1250

GU808261.1 CTCAGCAAAATCTCTTCTGTCCTGGAGTACTTCACGCACCACCAATTTAT 1250

GU808262.1 CTCAGCAAAATCTCTTCTGTCCTGGAGTACTTCACGCACCACCAATTTAT 1250

GU808263.1 CTCAGCAAAATCTCTTCTGTCCTGGAGTACTTCACGCACCACCAATTTAT 1250

GU808264.1 CTCGGCAAAATCTCTTCTGTCCTGGAGTACTTCACGCACCACCAATTTAT 1250

GU808265.1 CTCGGCAAAATCTCTTCTGTCCTGGAGTACTTCACGCACCACCAATTTAT 1250

L R K L S S V L E Y F T L R Q F L

GU808266.1 CTCAGAAAACTCTCTTCTGTCCTGGAGTACTTCACGCTCCGCCAGTTTCT 1250

GU808267.1 CTCAGAAAACTCTCTTCTGTCCTGGAGTACTTCACGCTCCGCCAGTTTCT 1250

GU808268.1 CTCAGAAAACTCTCTTCTGTCCTGGAGTACTTCACGCTCCGCCAGTTTCT 1250

GU808269.1 CTCAGAAAACTCTCTTCTGTCCTGGAGTACTTCACGCTCCGCCAGTTTCT 1250

GU808270.1 CTCAGAAAACTCTCTTCTGTCCTGGAGTACTTCACGCTCCGCCAGTTTCT 1250

GU808271.1 CTCAGAAAACTCTCTTCTGTCCTGGAGTACTTCACGCTCCGCCAGTTTCT 1250

GU808272.1 CTCAGAAAACTCTCTTCTGTCCTGGAGTACTTCACGCTCCGCCAGTTTCT 1250

GU808273.1 CTCAGAAAACTCTCTTCTGTCCTGGAGTACTTCACGCTCCGCCAGTTTCT 1250

GU808274.1 CTCAGAAAACTCTCTTCTGTCCTGGAGTACTTCACGCTCCGCCAGTTTCT 1250

GU808275.1 CTCAGAAAACTCTCTTCTGTCCTGGAGTACTTCACGCTCCGCCAGTTTCT 1250

GU808276.1 CTCAGAAAACTCTCTTCTGTCCTGGAGTACTTCACGCTCCGCCAGTTTCT 1250

F L D S N V R G L L R R M E G T D

GU808256.1 ATTCCTGGATAGCAACGTCAGAGGACTTCTCAGAAGGATGGAGGGCACAG 1300

GU808257.1 ATTCCTGGATAGCAACGTCAGAGGACTTCTCAGAAGGATGGAGGGCACAG 1300

GU808258.1 ATTCCTGGATAGCAACGTCAGAGGACTTCTCAGAAGGATGGAGGGCACAG 1300

GU808259.1 ATTCCTGGATAGCAACGTCAGAGGACTTCTCAGAAGGATGGAGGGCACAG 1300

GU808260.1 ATTCCTGGATAGCAACGTCAGAGGACTTCTCAGAAGGATGGAGGGCACAG 1300

GU808261.1 ATTCCTGGATAGCAACGTCAGAGGACTTCTCAGAAGGATGGAGGGCACAG 1300

GU808262.1 ATTCCTGGACAGCAACGTCAGAGGACTTCTCAGAAGGATGGAGGGCACAG 1300

GU808263.1 ATTCCTGGACAGCAACGTCAGAGGACTTCTCAGAAGGATGGAGGGCACAG 1300

GU808264.1 ATTCCTGGACAGCAACGTCAGAGGACTTCTCAGAAGGATGGAGGGCACAG 1300

GU808265.1 ATTCCTGGACAGCAACGTCAGAGGACTTCTCAGAAGGATGGAGGGCACAG 1300

F L D S N V R G L L R R M E G T D

GU808266.1 ATTCCTGGACAGCAACGTCAGAGGGCTTCTCAGAAGGATGGAGGGCACAG 1300

GU808267.1 ATTCCTGGACAGCAACGTCAGAGGGCTTCTCAGAAGGATGGAGGGCACAG 1300

GU808268.1 ATTCCTGGACAGCAACGTCAGAGGGCTTCTCAGAAGGATGGAGGGCACAG 1300

GU808269.1 ATTCCTGGACAGCAACGTCAGAGGGCTTCTCAGAAGGATGGAGGGCACAG 1300

GU808270.1 ATTCCTGGACAGCAACGTCAGAGGGCTTCTCAGAAGGATGGAGGGCACAG 1300

GU808271.1 ATTCCTGGATAGCAACGTCAGAGGGCTTCTCAGAAGGATGGAGGGCACAG 1300

GU808272.1 ATTCCTGGATAGCAACGTCAGAGGGCTTCTCAGAAGGATGGAGGGCACAG 1300

GU808273.1 ATTCCTGGACAGCAACGTCAGAGGGCTTCTCAGAAAGATGGAGGGCACAG 1300

GU808274.1 ATTCCTGGACAGCAACGTCAGAGGGCTTCTCAGAAAGATGGAGGGCACAG 1300

GU808275.1 ATTCCTGGACAGCAACGTCAGAGGGCTTCTCAGAAGGATGGAGGGCACAG 1300

GU808276.1 ATTCCTGGACAGCAACGTCAGAGGGCTTCTCAGAAGGATGGAGGGCACAG 1300

R Q T F N F D V T E I E W E P Y

GU808256.1 ACAGACAAACGTTTAATTTTGATGTCACTGAAATTGAGTGGGAGCCGTAT 1350

GU808257.1 ACAGACAAACGTTTAATTTTGATGTCACTGAAATTGAGTGGGAGCCGTAT 1350

GU808258.1 ACAGACAAACGTTTAATTTTGATGTCACTGAAATTGAGTGGGAGCCGTAT 1350

GU808259.1 ACAGACAAACGTTTAATTTTGATGTCACTGAAATTGAGTGGGAGCCGTAT 1350

GU808260.1 ACAGACAAACGTTTAATTTTGATGTCACTGAAATTGAGTGGGAGCCGTAT 1350

GU808261.1 ACAGACAAACGTTTAATTTTGATGTCACTGAAATTGAGTGGGAGCCGTAT 1350

GU808262.1 ACAGACAAACGTTTAATTTTGATGTCACTGAAATTGAGTGGGAGCCGTAT 1350

GU808263.1 ACAGACAAACGTTTAATTTTGATGTCACTGAAATTGAGTGGGAGCCGTAT 1350

GU808264.1 ACAGACAAACGTTTAATTTCGATGTCACTGAAATTGAGTGGGAGCCGTAT 1350

GU808265.1 ACAGACAAACGTTTAATTTCGATGTCACTGAAATTGAGTGGGAGCCGTAT 1350

R Q T F N F D V T E I E W E P F

GU808266.1 ACAGACAAACGTTTAATTTCGATGTCACTGAAATTGAGTGGGAGCCGTTT 1350

GU808267.1 ACAGACAAACGTTTAATTTCGATGTCGCTGAAATTGAGTGGGAGCCGTTT 1350

GU808268.1 ACAGACAAACGTTTAATTTCGATGTCACTGAAATTGAGTGGGAGCCGTTT 1350

GU808269.1 ACAGACAAACGTTTAATTTCGATGTCACTGAAATTGAGTGGGAGCCGTTT 1350

GU808270.1 ACAGACAAACGTTTAATTTCGATGTCACTGAAATTGAGTGGGAGCCGTTT 1350

GU808271.1 ACAGACAAACGTTTAATTTCGATGTCACTGAAATTGAGTGGGAGCCGTTT 1350

GU808272.1 ACAGGCAAACGTTTAATTTCGATGTCACTGAAATTGAGTGGGAGCCGTTT 1350

GU808273.1 ACAGACAAACGTTTAATTTCGATGTCACTGAAATTGAGTGGGAGCCGTTT 1350

GU808274.1 ACAGACAAACGTTTAATTTCGATGTCACTGAAATTGAGTGGGAGCCGTTT 1350

GU808275.1 ACAGACAAACGTTTAATTTCGATGTCACTGAAATTGAGTGGGAGCCGTTT 1350

GU808276.1 ACAGACAAACGTTTAATTTCGATGTCACTGAAATTGAGTGGGAGCCGTTT 1350

L Q N F V R G I A N N Y D Y S M *

GU808256.1 CTACAAAACTTTGTGCGCGGCATCGCAAATAATTACGACTATAGTATGTA 1400

GU808257.1 CTACAAAACTTTGTGCGCGGCATCGCAAATAATTACGACTATAGTATGTA 1400

GU808258.1 CTACAAAACTTTGTGCGCGGCATCGCAAATAATTACGACTATAGTATGTA 1400

GU808259.1 CTACAAAACTTTGTGCGCGGCATCGCAAATAATTACGACTATAGTATGTA 1400

GU808260.1 CTACAAAACTTTGTGCGCGGCATCGCAAATAATTACGACTATAGTATGTA 1400

GU808261.1 CTACAAAACTTTGTGCGCGGCATCGCAAATAATTACGACTATAGTATGTA 1400

GU808262.1 CTACAAAACTTTGTGCGCGGCATCGCAAATAATTACGACTATAGTATGTA 1400

GU808263.1 CTACAAAACTTTGTGCGCGGCATCGCAAATAATTACGACTATAGTATGTA 1400

GU808264.1 CTACAAAACTTTGTGCGCGGCATCGCAAATAATTACGACTATAGTATGTA 1400

GU808265.1 CTACAAAACTTTGTGCGCGGCATCGCAAATAATTACGACTATAGTATGTA 1400

L Q N C V R G I A N N Y D *

GU808266.1 CTACAAAATTGTGTGCGCGGCATCGCAAATAATTACGACTAGAGTAGGTA 1400

GU808267.1 CTACAAAATTGTGTGCGCGGCATCGCAAATAATTACGACTAGAGTAGGTA 1400

GU808268.1 CTACAAAATTGTGTGCGCGGCATCGCAAATAATTACGACTAGAGTAGGTA 1400

GU808269.1 CTACAAAATTGTGTGCGCGGCATCGCAAATAATTACGACTAGAGTAGGTA 1400

GU808270.1 CTACAAAATTGTGTGCGCGGCATCGCAAATAATTACGACTAGAGTAGGTA 1400

GU808271.1 CTACAAAATTGTGTGCGCGGCATCGCAAATAATTACGACTAGAGTAGGTA 1400

GU808272.1 CTACAAAATTGTGTGCGCGGCATCGCAAATAATTACGACTAGAGTAGGTA 1400

GU808273.1 CTACAAAATTGTGTGCGCGGCATCGCAAATAATTACGACTAGAGTAGGTA 1400

GU808274.1 CTACAAAATTGTGTGCGCGGCATCGCAAATAATTACGACTAGAGTAGGTA 1400

GU808275.1 CTACAAAATTGTGTGCGCGGCATCGCAAATAATTACGACTAGAGTAGGTA 1400

GU808276.1 CTACAAAATTGTGTGCGCGGCATCGCAAATAATTACGACTAGAGTAGGTA 1400

GU808256.1 ----ATATAGTATTGAGGTTTGATATTGATTGAGGAGAGGTGAAATTTAG 1446

GU808257.1 ----ATATAGTATTGAGGTTTGATATTGATTGAGGAGAGGTGAAATTTAG 1446

GU808258.1 ----ATATAGTATTGAGGTTTGATATTGATTGAGGAGAGGTGAAATTTAG 1446

GU808259.1 ----ATATAGTATTGAGGTTTGATATTGATTGAGGAGAGGTGAAATTTAG 1446

GU808260.1 ----ATATAGTATTGAGGTTTGATATTGATTGAGGAGAGGTGAAATTTAG 1446

GU808261.1 ----ATATAGTATTGAGGTTTGATATTGATTGAGGAGAGGTGAAATTTAG 1446

GU808262.1 ----ATATAGTATTGAGGTTTGATATTGATTGAGGAGAGGTGAAATTTAG 1446

GU808263.1 ----ATATAGTATTGAGGTTTGATATTGATTGAGGAGAGGTGAAATTTAG 1446

GU808264.1 ----ATATAGTATTGAGGTTTGATATTGATTGAGGAGAGGTGAAATTTAG 1446

GU808265.1 ----ATATAGTATTGAGGTTTGATATTGATTGAGGAGAGGTGAAATTTAG 1446

GU808266.1 TGTAATATAGTATTGAGGTTT-------------------------ATAG 1425

GU808267.1 TGTAATATAGTATTGAGGTTT-------------------------ATAG 1425

GU808268.1 TGTAATATAGTATTGAGGTTT-------------------------ATAG 1425

GU808269.1 TGTAATATAGTATTGAGGTTT-------------------------ATAG 1425

GU808270.1 TGTAATATAGTATTGAGGTTT-------------------------ATAG 1425

GU808271.1 TGTAATATAGTATTGAGGTTT-------------------------ATAG 1425

GU808272.1 TGTAATATAGTATTGAGGTTT-------------------------ATAG 1425

GU808273.1 TGTAATATAGTATTGAGGTTT-------------------------ATAG 1425

GU808274.1 TGTAATATAGTATTGAGGTTT-------------------------ATAG 1425

GU808275.1 TGTAATATAGTATTGAGGTTT-------------------------ATAG 1425

GU808276.1 TGTAATATAGTATTGAGGTTT-------------------------ATAG 1425

GU808256.1 CCTATGTTACTCGTGAATAATGTAGCTTTCGAATGGTGAAAGAATTATTA 1496

GU808257.1 CCTATGTTACTCGTGAATAATGTAGCTTTCGAATGGTGAAAGAATTATTA 1496

GU808258.1 CCTATGTTACTCGTGAATAATGTAGCTTTCGAATGGTGAAAGAATTATTA 1496

GU808259.1 CCTATGTTACTCGTGAATAATGTAGCTTTCGAATGGTGAAAGAATTATTA 1496

GU808260.1 CCTATGTTACTCGTGAATAATGTAGCTTTCGAATGGTGAAAGAATTATTA 1496

GU808261.1 CCTATGTTACTCGTGAATAATGTAGCTTTCGAATGGTGAAAGAATTATTA 1496

GU808262.1 CCTATGTTACTCGTGAATAATGTAGCTTTCGAATGGTGAAAGAATTATTA 1496

GU808263.1 CCTATGTTACTCGTGAATAATGTAGCTTTCGAATGGTGAAAGAATTATTA 1496

GU808264.1 CCTATGTTACTCGTGAATAATGTAGCTTTCGAATGGTGAAAGAATTATTA 1496

GU808265.1 CCTATGTTACTCGTGAATAATGTGGCTTTCGAATGGTGAAAGAATTATTA 1496

GU808266.1 CCTTG---------GACCAATAAAGCCTAG--ATG--GATAGTCTTCATA 1462

GU808267.1 CCTTG---------GACCAATAAAGCCTAG--ATA--GATAGTCTTCATA 1462

GU808268.1 CCTTG---------GACCAATAAAGCCTAG--ATA--GATAGTCTTCATA 1462

GU808269.1 CCTTG---------GACCAATAAAGCCTAG--ATG--GATAGTCTTCATA 1462

GU808270.1 CCTTG---------GACCAATAAAGCCTAG--ATA--GATAGTCTTCATA 1462

GU808271.1 CCTTG---------GACCAATAAAGCCTAG--ATG--GATAGTCTTCATA 1462

GU808272.1 CCTTG---------GACCAATAAAGCCTAG--ATG--GATAGTCTTCATA 1462

GU808273.1 CCTTG---------GACCAATAAAGCCTAG--ATG--GATAGTCTTCATA 1462

GU808274.1 CCTTG---------GACCAATAAAGCCTAG--ATG--GATAGTCTTCATA 1462

GU808275.1 CCTTG---------GACCAATAAAGCCTAG--ATG--GATAGTCTTCATA 1462

GU808276.1 CCTTG---------GACCAATAAAGCCTAG--ATA--GATAGTCTTCATA 1462

GU808256.1 AAAACGGTCCAGTAGTTTTTGAGCCTATAAATTACAACCAAACAAA---C 1543

GU808257.1 AAAACGGTCCAGTAGTTTTTGAGCCTATAAATTACAACCAAACAAA---C 1543

GU808258.1 AAAACGGTCCAGTAGTTTTTGAGCCTATAAATTACAACCAAACAAA---C 1543

GU808259.1 AAAACGGTCCAGTAGTTTTTGAGCCTATAAATTACAACCAAACAAA---C 1543

GU808260.1 AAAACGGTCCAGTAGTTTTTGAGCCTATAAATTACAACCAAACAAA---C 1543

GU808261.1 AAAACGGTCCAGTAGTTTTTGAGCCTATAAATTACAACCAAACAAA---C 1543

GU808262.1 AAAACGGTCCAGTAGTTTTTGAGCCTATAAATTACAACCAAACAAA---C 1543

GU808263.1 AAAACGGTCCAGTAGTTTTTGAGCCTATAAATTACAACCAAACAAA---C 1543

GU808264.1 AAAACGGTCCAGTAGTTTTTGAGCCTATTAATTACAACCAAACAAACTTC 1546

GU808265.1 AAAACGGTCCAGTAGTTTTTGAGCCTATTAATTACAACCAAACAAACTTC 1546

GU808266.1 CAAACG--------------------------------CTTACTCA---- 1476

GU808267.1 CAAACG--------------------------------CTTACTCA---- 1476

GU808268.1 CAAACG--------------------------------CTTACTCA---- 1476

GU808269.1 CAAACG--------------------------------CTTACTCA---- 1476

GU808270.1 CAAACG--------------------------------CTTACTCA---- 1476

GU808271.1 CAAACG--------------------------------CTTACTCA---- 1476

GU808272.1 CAAACG--------------------------------CTTACTCA---- 1476

GU808273.1 CAAACG--------------------------------CTTACTCA---- 1476

GU808274.1 CAAACG--------------------------------CTTACTCA---- 1476

GU808275.1 CAAACG--------------------------------CTTACTCA---- 1476

GU808276.1 CAAACG--------------------------------CTTACTCG---- 1476

GU808256.1 CTAATAGTAGTGTAGATTTGAATTTGAAAAGTGAGGTATAAAATCTTGTC 1593

GU808257.1 CTAATAGTAGTGTAGATTTGAATTTGAAAAGTGAGGTATAAAATCTTGTC 1593

GU808258.1 CTAATAGTAGTGTAGATTTGAATTTGAAAAGTGAGGTATAAAATCTTGTC 1593

GU808259.1 CTAATAGTAGTGTAGATTTGAATTTGAAAAGTGAGGTATAAAATCTTGTC 1593

GU808260.1 CTAATAGTAGTGTAGATTTGAATTTGAAAAGTGAGGTATAAAATCTTGTC 1593

GU808261.1 CTAATAGTAGTGTAGATTTGAATTTGAAAAGTGAGGTATAAAATCTTGTC 1593

GU808262.1 CTAATATTAGTGTAGATTTGAATTTGAAAAGTGAGGTATAAAATCTTGTC 1593

GU808263.1 CTAATATTAGTGTAGATTTGAATTTGAAAAGTGAGGTATAAAATCTTGTC 1593

GU808264.1 CTAATATTAGTGTAGATTTGAATTTGAAAAGTGAGGTATAAAATCTTGTC 1596

GU808265.1 CTAATATTAGTGTAGATTTGAATTTGAAAAGTGAGGTATAAAATCTTGTC 1596

GU808266.1 ---------------------------AAAGTGAGGTATAAAATCTTGTC 1499

GU808267.1 ---------------------------AAAGTGAGGTATAAAATCTTGTC 1499

GU808268.1 ---------------------------AAAGTGAGGTATAAAATCTTGTC 1499

GU808269.1 ---------------------------AAAGTGAGGTATAAAATCTTGTC 1499

GU808270.1 ---------------------------AAAGTGAGGTATAAAATCTTGTC 1499

GU808271.1 ---------------------------AAAGTGAGGTATAAAATCTTGTC 1499

GU808272.1 ---------------------------AAAGTGAGGTATAAAATCTTGTC 1499

GU808273.1 ---------------------------AAAGTGAGGTATAAAATCTTGTC 1499

GU808274.1 ---------------------------AAAGTGAGGTATAAAATCTTGTC 1499

GU808275.1 ---------------------------AAAGTGAGGTATAAAATCTTGTC 1499

GU808276.1 ---------------------------AAAGTGAGGTATAAAATCTTGTC 1499

GU808256.1 ATTACTG 1600

GU808257.1 ATTACTG 1600

GU808258.1 ATTACTG 1600

GU808259.1 ATTACTG 1600

GU808260.1 ATTACTG 1600

GU808261.1 ATTACTG 1600

GU808262.1 ATTACTG 1600

GU808263.1 ATTACTG 1600

GU808264.1 ATTACTG 1603

GU808265.1 ATTACTG 1603

GU808266.1 ATTACTG 1506

GU808267.1 ATTACTG 1506

GU808268.1 ATTACTG 1506

GU808269.1 ATTACTG 1506

GU808270.1 ATTACTG 1506

GU808271.1 ATTACTG 1506

GU808272.1 ATTACTG 1506

GU808273.1 ATTACTG 1506

GU808274.1 ATTACTG 1506

GU808275.1 ATTACTG 1506

GU808276.1 ATTACTG 1506
