# Supplementary material for: Frequency of hybridization between Ostrinia nubilalis E-and Z-pheromone races in regions of sympatry within the United States
Source: Ecol Evol. 2013 Jun 24;3(8):2459–70. doi: 10.1002/ece3.639 (PMC3930039; doi:10.1002/ece3.639)

**Figure S2.** Restriction fragment length polymorphism observed following agarose gel electrophoresis of *Taq*I, *NdeI*I and *Mse*I digested PCR fragements (see Materials and Methods). The pgfar-e and pgfar-z alleles are indicated by the corresponding arrows, and heterozygotes are labeled with an "H".


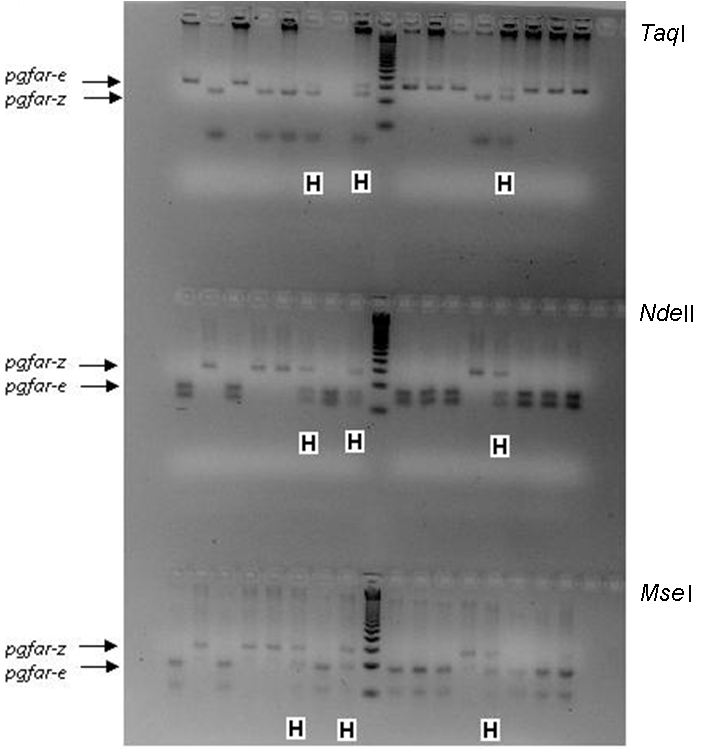

Supplement: Supplementary file 2 [file ece30003-2459-SD2.doc]
