# Supplementary material for: Frequency of hybridization between Ostrinia nubilalis E-and Z-pheromone races in regions of sympatry within the United States
Source: Ecol Evol. 2013 Jun 24;3(8):2459–70. doi: 10.1002/ece3.639 (PMC3930039; doi:10.1002/ece3.639)
Supplement: Supplementary file 3 — Table S1. The pgfar genotypes and corresponding gas chromatograph (GC)-determined proportion of E11-14:OAc among females from Families 1–6 [file ece30003-2459-SD3.doc]

**Table S1.** Paired *pgfar* SNP genotype and *E*-Δ11-tetradecenyl acetate titre estimates for phenotype among 269 female *O. nubilalis*.

|  |  |  | SNP marker assays | | |  |  |
| --- | --- | --- | --- | --- | --- | --- | --- |
| Female ID | Family | Family  Individual | *pgfar*  *Taq*I | *pgfar*  *Nde*II | *pgfar*  *Mse*I | Phenotype | *E*-titre |
| F001 | 1 | 11 | Z | Z | Z | Z | 2 |
| F002 | 1 | 12 | Z | Z | Z | Z | 2 |
| F003 | 1 | 13 | H | H | H | H | 61 |
| F004 | 1 | 14 | Z | Z | Z | Z | 3 |
| F005 | 1 | 15 | Z | H | H | H | 68 |
| F006 | 1 | 16 | Z | Z | Z | Z | 2 |
| F007 | 1 | 17 | Z | Z | Z | Z | 2 |
| F008 | 1 | 18 | H | E | E | H | 94 |
| F009 | 1 | 19 | E | E | E | E | 99 |
| F010 | 1 | 20 | Z | Z | Z | Z | 2 |
| F011 | 1 | 21 | Z | Z | Z | Z | 2 |
| F012 | 1 | 22 | H | H | H | H | 59 |
| F013 | 1 | 23 | H | H | H | H | 64 |
| F014 | 1 | 24 | Z | Z | Z | Z | 2 |
| F015 | 1 | 25 | Z | Z | Z | Z | 2 |
| F016 | 1 | 26 | Z | Z | Z | Z | 2 |
| F017 | 1 | 27 | Z | Z | Z | Z | 2 |
| F018 | 1 | 28 | E | E | E | E | 99 |
| F019 | 1 | 29 | H | H | H | H | 70 |
| F020 | 1 | 30 | E | E | E | E | 99 |
| F021 | 1 | 31 | E | E | E | E | 99 |
| F022 | 1 | 32 | Z | Z | Z | Z | 3 |
| F023 | 1 | 33 | H | H | H | H | 53 |
| F024 | 1 | 35 | Z | Z | Z | Z | 2 |
| F025 | 1 | 36 | Z | Z | Z | Z | 2 |
| F026 | 1 | 37 | E | E | E | E | 99 |
| F027 | 1 | 38 | E | E | E | U | NA |
| F028 | 1 | 39 | E | E | E | E | 99 |
| F029 | 1 | 40 | Z | Z | Z | Z | 2 |
| F030 | 1 | 41 | Z | Z | Z | Z | 2 |
| F031 | 1 | 42 | H | H | H | H | 70 |
| F032 | 1 | 43 | Z | H | H | H | 57 |
| F033 | 1 | 44 | Z | Z | Z | Z | 2 |
| F034 | 1 | 45 | Z | H | H | H | 67 |
| F035 | 1 | 46 | H | H | H | H | 63 |
| F036 | 1 | 47 | Z | Z | Z | Z | 3 |
| F037 | 1 | 48 | H | ? | ? | H | 69 |
| F038 | 1 | 49 | E | E | E | E | 99 |
|  |  |  | SNP marker assays | | |  |  |
| Female ID | Family | Family  Individual | *pgfar*  *Taq*I | *pgfar*  *Nde*II | *pgfar*  *Mse*I | Phenotype | *E*-titre |
| F039 | 1 | 50 | H | H | H | H | 60 |
| F040 | 1 | 51 | Z | H | H | H | 65 |
| F041 | 1 | 52 | Z | Z | Z | Z | 2 |
| F042 | 1 | 53 | H | H | H | H | 80 |
| F043 | 1 | 54 | H | H | H | H | 66 |
| F044 | 1 | 55 | H | H | H | H | 55 |
| F045 | 1 | 57 | Z | Z | Z | Z | 2 |
| F046 | 1 | 58 | Z | Z | Z | Z | 2 |
| F047 | 1 | 59 | Z | Z | Z | Z | 2 |
| F048 | 2 | 11 | Z | Z | Z | Z | 2 |
| F049 | 2 | 12 | Z | Z | Z | Z | 3 |
| F050 | 2 | 13 | H | H | H | H | 55 |
| F051 | 2 | 14 | Z | Z | Z | Z | 2 |
| F052 | 2 | 15 | H | H | H | H | 66 |
| F053 | 2 | 16 | Z | Z | Z | Z | 2 |
| F054 | 2 | 17 | Z | Z | Z | Z | 2 |
| F055 | 2 | 18 | Z | Z | Z | Z | 2 |
| F056 | 2 | 19 | Z | Z | Z | Z | 2 |
| F057 | 2 | 20 | Z | Z | Z | Z | 2 |
| F058 | 2 | 21 | Z | Z | Z | Z | 3 |
| F059 | 2 | 22 | H | H | H | H | 64 |
| F060 | 2 | 23 | Z | Z | Z | Z | 2 |
| F061 | 2 | 24 | Z | Z | Z | Z | 4 |
| F062 | 2 | 25 | Z | Z | Z | Z | 2 |
| F063 | 2 | 26 | Z | Z | Z | Z | 2 |
| F064 | 2 | 27 | Z | Z | Z | Z | 2 |
| F065 | 2 | 28 | Z | Z | Z | Z | 2 |
| F066 | 2 | 29 | Z | Z | Z | U | NA |
| F067 | 2 | 30 | Z | Z | Z | Z | 2 |
| F068 | 2 | 32 | Z | Z | Z | Z | 2 |
| F069 | 2 | 33 | Z | Z | Z | Z | 3 |
| F070 | 2 | 34 | Z | Z | Z | Z | 2 |
| F071 | 2 | 35 | Z | Z | Z | Z | 2 |
| F072 | 2 | 36 | Z | Z | Z | Z | 2 |
| F073 | 2 | 37 | Z | Z | Z | Z | 2 |
| F074 | 2 | 38 | Z | Z | Z | Z | 2 |
| F075 | 2 | 39 | Z | Z | Z | Z | 3 |
| F076 | 2 | 40 | Z | Z | Z | Z | 2 |
| F077 | 2 | 41 | Z | Z | Z | Z | 2 |
| F078 | 2 | 42 | Z | Z | Z | Z | 3 |
| F079 | 2 | 43 | Z | Z | Z | Z | 2 |
| F080 | 2 | 44 | Z | Z | Z | U | NA |
|  |  |  | SNP marker assays | | |  |  |
| Female ID | Family | Family  Individual | *pgfar*  *Taq*I | *pgfar*  *Nde*II | *pgfar*  *Mse*I | Phenotype | *E*-titre |
| F081 | 2 | 45 | Z | Z | Z | Z | 2 |
| F082 | 2 | 46 | Z | Z | Z | Z | 2 |
| F083 | 2 | 47 | H | H | H | H | 67 |
| F084 | 2 | 48 | Z | Z | Z | Z | 2 |
| F085 | 2 | 49 | Z | Z | Z | Z | 4 |
| F086 | 2 | 50 | Z | Z | Z | Z | 2 |
| F087 | 2 | 51 | Z | Z | Z | Z | 2 |
| F088 | 2 | 52 | Z | Z | Z | Z | 2 |
| F089 | 2 | 53 | Z | Z | Z | Z | 2 |
| F090 | 2 | 54 | Z | Z | Z | Z | 3 |
| F091 | 2 | 55 | Z | Z | Z | Z | 2 |
| F092 | 2 | 56 | Z | Z | Z | Z | 2 |
| F093 | 2 | 57 | H | H | H | H | 53 |
| F094 | 2 | 58 | Z | Z | Z | Z | 2 |
| F095 | 2 | 59 | Z | Z | Z | Z | 2 |
| F096 | 3 | 11 | Z | Z | Z | Z | 2 |
| F097 | 3 | 12 | Z | Z | Z | Z | 2 |
| F098 | 3 | 13 | H | H | H | H | 62 |
| F099 | 3 | 14 | Z | Z | Z | Z | 2 |
| F100 | 3 | 15 | H | H | H | H | 94 |
| F101 | 3 | 16 | E | E | E | E | 99 |
| F102 | 3 | 17 | Z | Z | Z | Z | 2 |
| F103 | 3 | 18 | Z | Z | Z | Z | 2 |
| F104 | 3 | 19 | Z | Z | Z | Z | 2 |
| F105 | 3 | 20 | Z | Z | Z | Z | 3 |
| F106 | 3 | 21 | E | E | E | E | 99 |
| F107 | 3 | 22 | Z | Z | Z | Z | 2 |
| F108 | 3 | 23 | H | H | H | H | 68 |
| F109 | 3 | 24 | Z | Z | Z | Z | 2 |
| F110 | 3 | 25 | H | H | H | H | 75 |
| F111 | 3 | 27 | Z | Z | Z | Z | 2 |
| F112 | 3 | 28 | Z | Z | Z | U | NA |
| F113 | 3 | 29 | Z | Z | Z | Z | 3 |
| F114 | 3 | 30 | Z | Z | Z | Z | 2 |
| F115 | 3 | 31 | Z | Z | Z | Z | 2 |
| F116 | 3 | 32 | Z | Z | Z | Z | 3 |
| F117 | 3 | 33 | Z | Z | Z | Z | 2 |
| F118 | 3 | 34 | H | H | H | H | 64 |
| F119 | 3 | 35 | E | E | E | H | 82 |
| F120 | 3 | 36 | Z | Z | Z | Z | 2 |
| F121 | 3 | 37 | E | E | E | E | 97 |
| F122 | 3 | 38 | H | H | H | H | 68 |
|  |  |  | SNP marker assays | | |  |  |
| Female ID | Family | Family  Individual | *pgfar*  *Taq*I | *pgfar*  *Nde*II | *pgfar*  *Mse*I | Phenotype | *E*-titre |
| F123 | 3 | 39 | H | H | H | H | 66 |
| F124 | 3 | 40 | H | H | H | H | 79 |
| F125 | 3 | 41 | Z | Z | Z | Z | 2 |
| F126 | 3 | 42 | Z | Z | Z | U | NA |
| F127 | 3 | 43 | Z | Z | Z | Z | 2 |
| F128 | 3 | 44 | Z | Z | Z | Z | 2 |
| F129 | 3 | 45 | Z | Z | Z | Z | 2 |
| F130 | 3 | 47 | Z | Z | Z | Z | 2 |
| F131 | 3 | 48 | H | H | H | H | 94 |
| F132 | 3 | 49 | Z | Z | Z | Z | 11 |
| F133 | 3 | 50 | Z | Z | Z | Z | 2 |
| F134 | 3 | 51 | H | H | H | H | 89 |
| F135 | 3 | 52 | Z | Z | Z | Z | 2 |
| F136 | 3 | 53 | H | H | H | H | 86 |
| F137 | 3 | 54 | E | E | E | E | 99 |
| F138 | 3 | 55 | Z | Z | Z | Z | 2 |
| F139 | 3 | 56 | H | H | H | H | 74 |
| F140 | 3 | 57 | E | E | E | E | 99 |
| F141 | 3 | 58 | Z | Z | Z | Z | 2 |
| F142 | 3 | 59 | E | E | E | E | 99 |
| F143 | 3 | 60 | Z | Z | Z | Z | 2 |
| F144 | 4 | 11 | Z | Z | Z | U | NA |
| F145 | 4 | 12 | Z | Z | Z | Z | 2 |
| F146 | 4 | 14 | Z | Z | Z | Z | 2 |
| F147 | 4 | 15 | H | H | H | H | 60 |
| F148 | 4 | 16 | Z | Z | Z | U | NA |
| F149 | 4 | 17 | Z | Z | Z | Z | 2 |
| F150 | 4 | 18 | E | E | E | E | 99 |
| F151 | 4 | 19 | Z | Z | Z | Z | 2 |
| F152 | 4 | 21 | Z | Z | Z | Z | 2 |
| F153 | 4 | 22 | Z | Z | Z | Z | 2 |
| F154 | 4 | 23 | Z | Z | Z | Z | 2 |
| F155 | 4 | 24 | Z | Z | Z | Z | 2 |
| F156 | 4 | 25 | H | H | H | H | 72 |
| F157 | 4 | 26 | Z | Z | Z | H | 76 |
| F158 | 4 | 27 | Z | Z | Z | Z | 2 |
| F159 | 4 | 28 | Z | Z | Z | Z | 2 |
| F160 | 4 | 29 | Z | Z | Z | Z | 2 |
| F161 | 4 | 30 | Z | Z | Z | Z | 2 |
| F162 | 4 | 31 | Z | Z | Z | Z | 2 |
| F163 | 4 | 32 | Z | Z | Z | Z | 2 |
| F164 | 4 | 33 | H | H | H | H | 82 |
|  |  |  | SNP marker assays | | |  |  |
| Female ID | Family | Family  Individual | *pgfar*  *Taq*I | *pgfar*  *Nde*II | *pgfar*  *Mse*I | Phenotype | *E*-titre |
| F165 | 4 | 35 | Z | Z | Z | Z | 2 |
| F166 | 4 | 36 | ? | ? | ? | H | 67 |
| F167 | 4 | 38 | Z | Z | Z | Z | 2 |
| F168 | 4 | 39 | H | H | H | H | 67 |
| F169 | 4 | 40 | H | H | H | H | 67 |
| F170 | 4 | 41 | Z | Z | Z | Z | 2 |
| F171 | 4 | 42 | Z | Z | Z | Z | 3 |
| F172 | 4 | 43 | Z | Z | Z | Z | 2 |
| F173 | 4 | 45 | Z | Z | Z | Z | 2 |
| F174 | 4 | 46 | H | H | H | H | 60 |
| F175 | 4 | 49 | H | H | H | H | 76 |
| F176 | 5 | 3 | Z | Z | Z | Z | 2 |
| F177 | 5 | 4 | ? | Z | Z | Z | 3 |
| F178 | 5 | 6 | H | H | H | H | 68 |
| F179 | 5 | 10 | H | H | H | H | 72 |
| F180 | 5 | 16 | ? | Z | Z | Z | 2 |
| F181 | 5 | 22 | ? | E | E | E | 99 |
| F182 | 5 | 25 | H | H | H | H | 68 |
| F183 | 5 | 27 | E | E | E | E | 99 |
| F184 | 5 | 28 | E | E | E | E | 99 |
| F185 | 5 | 34 | Z | H | H | H | 66 |
| F186 | 5 | 35 | E | E | E | E | 99 |
| F187 | 5 | 36 | H | H | H | H | 58 |
| F188 | 5 | 37 | Z | Z | Z | Z | 2 |
| F189 | 5 | 38 | E | E | E | E | 99 |
| F190 | 5 | 39 | H | H | H | H | 56 |
| F191 | 5 | 41 | Z | Z | Z | Z | 2 |
| F192 | 5 | 45 | ? | E | E | H | 77 |
| F193 | 5 | 46 | E | H | H | H | 72 |
| F194 | 5 | 48 | H | H | H | H | 72 |
| F195 | 5 | 50 | E | E | E | E | 99 |
| F196 | 5 | 52 | H | H | H | H | 64 |
| F197 | 5 | 53 | Z | Z | Z | Z | 2 |
| F198 | 5 | 54 | H | H | H | H | 63 |
| F199 | 5 | 55 | E | E | E | E | 99 |
| F200 | 5 | 56 | H | H | H | H | 72 |
| F201 | 5 | 57 | H | H | H | H | 57 |
| F202 | 6 | 5 | H | H | H | H | 69 |
| F203 | 6 | 8 | Z | Z | Z | Z | 2 |
| F204 | 6 | 10 | Z | Z | Z | Z | 2 |
| F205 | 6 | 11 | Z | Z | Z | Z | 2 |
| F206 | 6 | 12 | Z | Z | Z | Z | 2 |
|  |  |  | SNP marker assays | | |  |  |
| Female ID | Family | Family  Individual | *pgfar*  *Taq*I | *pgfar*  *Nde*II | *pgfar*  *Mse*I | Phenotype | *E*-titre |
| F207 | 6 | 13 | Z | Z | Z | U | NA |
| F208 | 6 | 14 | Z | Z | Z | Z | 2 |
| F209 | 6 | 16 | Z | Z | Z | Z | 2 |
| F210 | 6 | 17 | Z | Z | Z | U | NA |
| F211 | 6 | 19 | H | H | H | H | 64 |
| F212 | 6 | 20 | E | E | E | E | 99 |
| F213 | 6 | 21 | H | H | H | H | 63 |
| F214 | 6 | 22 | Z | Z | Z | Z | 2 |
| F215 | 6 | 23 | H | H | H | H | 58 |
| F216 | 6 | 25 | Z | Z | Z | Z | 2 |
| F217 | 6 | 26 | H | H | H | H | 65 |
| F218 | 6 | 27 | Z | Z | Z | Z | 2 |
| F219 | 6 | 29 | H | H | H | H | 60 |
| F220 | 6 | 30 | H | H | H | H | 71 |
| F221 | 6 | 31 | H | H | H | H | 64 |
| F222 | 6 | 32 | Z | Z | Z | Z | 2 |
| F223 | E | 12 | E | E | E | E | 99 |
| F224 | E | 13 | E | E | E | E | 99 |
| F225 | E | 16 | E | E | E | E | 99 |
| F226 | E | 17 | E | E | E | E | 99 |
| F227 | E | 18 | E | E | E | E | 99 |
| F228 | E | 20 | ? | E | E | E | 99 |
| F229 | E | 21 | E | E | E | E | 99 |
| F230 | E | 22 | E | E | E | E | 99 |
| F231 | E | 23 | E | E | E | E | 99 |
| F232 | E | 24 | E | E | E | E | 99 |
| F233 | E | 25 | E | E | E | E | 99 |
| F234 | E | 26 | E | E | E | U | NA |
| F235 | E | 27 | E | E | E | E | 99 |
| F236 | E | 28 | E | E | E | E | 99 |
| F237 | E | 29 | E | E | E | E | 99 |
| F238 | E | 30 | E | E | E | E | 99 |
| F239 | E | 31 | E | E | E | E | 99 |
| F240 | E | 32 | E | E | E | E | 99 |
| F241 | E | 33 | E | E | E | E | 99 |
| F242 | E | 34 | E | E | E | E | 99 |
| F243 | E | 35 | E | E | E | E | 99 |
| F244 | E | 36 | E | E | E | E | 99 |
| F245 | E | 37 | E | E | E | E | 99 |
| F246 | Z | 3 | Z | Z | Z | U | NA |
| F247 | Z | 4 | Z | Z | Z | Z | 2 |
| F248 | Z | 6 | Z | Z | Z | Z | 2 |
|  |  |  | SNP marker assays | | |  |  |
| Female ID | Family | Family  Individual | *pgfar*  *Taq*I | *pgfar*  *Nde*II | *pgfar*  *Mse*I | Phenotype | *E*-titre |
| F249 | Z | 7 | Z | Z | Z | Z | 2 |
| F250 | Z | 8 | Z | Z | Z | Z | 2 |
| F251 | Z | 9 | Z | Z | Z | U | NA |
| F252 | Z | 10 | Z | Z | Z | Z | 2 |
| F253 | Z | 11 | Z | Z | Z | Z | 2 |
| F254 | Z | 12 | Z | Z | Z | U | NA |
| F255 | Z | 13 | Z | Z | Z | Z | 2 |
| F256 | Z | 14 | Z | Z | Z | U | NA |
| F257 | Z | 15 | Z | Z | Z | Z | 2 |
| F258 | Z | 16 | Z | Z | Z | Z | 7 |
| F259 | Z | 17 | Z | Z | Z | Z | 2 |
| F260 | Z | 18 | Z | Z | Z | U | NA |
| F261 | Z | 19 | Z | Z | Z | Z | 2 |
| F262 | Z | 20 | Z | Z | Z | Z | 2 |
| F263 | Z | 21 | Z | Z | Z | U | NA |
| F264 | Z | 22 | Z | Z | Z | U | NA |
| F265 | Z | 23 | Z | Z | Z | Z | 2 |
| F266 | Z | 24 | Z | Z | Z | Z | 2 |
| F267 | Z | 26 | Z | Z | Z | U | NA |
| F268 | Z | 27 | Z | Z | Z | Z | 2 |
| F269 | Z | 28 | Z | Z | Z | Z | 2 |
