# Supplementary material for: Frequency of hybridization between Ostrinia nubilalis E-and Z-pheromone races in regions of sympatry within the United States
Source: Ecol Evol. 2013 Jun 24;3(8):2459–70. doi: 10.1002/ece3.639 (PMC3930039; doi:10.1002/ece3.639)
Supplement: Supplementary file 4 — Table S2. Results of hierarchical population genetic structure using analysis of molecular variance (AMOVA.). [file ece30003-2459-SD4.doc]

**Table S2.** Analysis of Molecular Variance (AMOVA) between groups (homogenous Z-pheromone race populations at sites 1 to 5 verses sympatric sites 5 to 16; Fig. 1) based upon genotypic data collected using *pgfar* SNP markers.

----------------------------------------------------------------------

Source of Sum of Variance Percentage

variation d.f. squares components of variation

----------------------------------------------------------------------

Among

groups 1 10.024 0.02526 Va 7.82

Among

populations

within

groups 14 15.439 0.01342 Vb 4.16

Among

individuals

within

populations 435 162.331 0.08903 Vc 27.58

Within

individuals 451 88.000 0.19512 Vd 60.44

----------------------------------------------------------------------

Total 901 275.794 0.32283

----------------------------------------------------------------------

Fixation Indices

FIS : 0.31331

FSC : 0.04510

FCT : 0.07824

FIT : 0.39558

----------------------------------------------------------------------

Significance tests (1023 permutations)

------------------

Vd and FIT : P(rand. value < obs. value) = 0.00000

P(rand. value = obs. value) = 0.00000

P-value = 0.00000+-0.00000

Vc and FIS : P(rand. value > obs. value) = 0.00000

P(rand. value = obs. value) = 0.00000

P-value = 0.00000+-0.00000

Vb and FSC : P(rand. value > obs. value) = 0.00293

P(rand. value = obs. value) = 0.00000

P-value = 0.00293+-0.00164

Va and FCT : P(rand. value > obs. value) = 0.01173

P(rand. value = obs. value) = 0.00000

P-value = 0.01173+-0.00363
