# Supplementary material for: Frequency of hybridization between Ostrinia nubilalis E-and Z-pheromone races in regions of sympatry within the United States
Source: Ecol Evol. 2013 Jun 24;3(8):2459–70. doi: 10.1002/ece3.639 (PMC3930039; doi:10.1002/ece3.639)
Supplement: Supplementary file 5 [file ece30003-2459-SD5.doc]

**Table S3.** Pairwise *F*_ST_ estimates between North American populations (below diagonal) and associated *P*-values (above diagonal). The codes for each location (1 through 16) are given in Figure 1.

Locations

|  | 1 | 2 | 3 | 4 | 5 | 6 | 7 | 8 | 9 | 10 | 11 | 12 | 13 | 14 | 15 | 16 |
| --- | --- | --- | --- | --- | --- | --- | --- | --- | --- | --- | --- | --- | --- | --- | --- | --- |
| 1 |  | 0.999 | 0.999 | 0.999 | 0.999 | 0.229 | 0.000 | 0.004 | 0.019 | 0.006 | 0.190 | 0.002 | 0.740 | 0.337 | 0.102 | 0.999 |
| 2 | 0.000 |  | 0.999 | 0.999 | 0.244 | .0232 | 0.000 | 0.003 | 0.017 | 0.004 | 0.162 | 0.000 | 0.422 | 0.186 | 0.097 | 0.999 |
| 3 | 0.000 | 0.000 |  | 0.999 | 0.242 | 0.237 | 0.001 | 0.004 | 0.024 | 0.003 | 0.166 | 0.000 | 0.412 | 0.179 | 0.079 | 0.999 |
| 4 | 0.000 | 0.000 | 0.000 |  | 0.235 | 0.238 | 0.000 | 0.003 | 0.017 | 0.005 | 0.166 | 0.000 | 0.411 | 0.198 | 0.098 | 0.999 |
| 5 | 0.027 | 0.043 | 0.043 | 0.043 |  | 0.999 | 0.036 | 0.045 | 0.194 | 0.010 | 0.999 | 0.008 | 0.638 | 0.999 | 0.999 | 0.524 |
| 6 | 0.048 | 0.064 | 0.064 | 0.064 | -0.018 |  | 0.091 | 0.087 | 0.241 | 0.241 | 0.761 | 0.019 | 0.469 | 0.755 | 0.999 | 0.479 |
| 7 | 0.161 | 0.175 | 0.175 | 0.175 | 0.066 | 0.042 |  | 0.873 | 0.879 | 0.879 | 0.002 | 0.148 | 0.127 | 0.029 | 0.270 | 0.017 |
| 8 | 0.134 | 0.144 | 0.144 | 0.144 | 0.068 | 0.048 | -0.010 |  | 0.356 | 0.761 | 0.008 | 0.156 | 0.041 | 0.031 | 0.277 | 0.055 |
| 9 | 0.105 | 0.117 | 0.117 | 0.117 | 0.031 | 0.013 | -0.007 | 0.002 |  | 0.674 | 0.080 | 0.082 | 0.174 | 0.163 | 0.600 | 0.175 |
| 10 | 0.172 | 0.186 | 0.186 | 0.186 | 0.063 | 0.037 | -0.017 | -0.011 | -0.010 |  | 0.021 | 0.192 | 0.063 | 0.087 | 0.364 | 0.034 |
| 11 | 0.021 | 0.033 | 0.033 | 0.033 | -0.016 | -0.012 | 0.085 | 0.081 | 0.042 | 0.082 |  | 0.000 | 0.674 | 0.999 | 0.628 | 0.365 |
| 12 | 0.498 | 0.516 | 0.516 | 0.516 | 0.314 | 0.262 | 0.059 | 0.055 | 0.124 | 0.065 | 0.372 |  | 0.010 | 0.007 | 0.091 | 0.015 |
| 13 | -0.008 | 0.010 | 0.010 | 0.010 | -0.014 | 0.000 | 0.102 | 0.096 | 0.060 | 0.102 | -0.009 | 0.367 |  | 0.999 | 0.551 | 0.999 |
| 14 | 0.016 | 0.033 | 0.033 | 0.033 | -0.022 | -0.015 | 0.078 | 0.077 | 0.040 | 0.075 | -0.017 | 0.330 | -0.022 |  | 0.598 | 0.522 |
| 15 | 0.085 | 0.108 | 0.108 | 0.108 | -0.028 | -0.034 | 0.017 | 0.027 | -0.006 | 0.012 | -0.022 | 0.194 | -0.001 | -0.022 |  | 0.477 |
| 16 | 0.000 | 0.000 | 0.000 | 0.000 | 0.014 | 0.030 | 0.126 | 0.119 | 0.085 | 0.129 | 0.012 | 0.382 | -0.014 | 0.004 | 0.048 |  |
